# Supplementary material for: Bird species define the relationship between West Nile viremia and infectiousness to Culex pipiens mosquitoes
Source: PLoS Negl Trop Dis. 2022 Oct 6;16(10):e0010835. doi: 10.1371/journal.pntd.0010835 (PMC9578590; doi:10.1371/journal.pntd.0010835)
Supplement: S1 Text — Table A. Information on birds and viremias as determined by plaque assay of engorged mosquitoes frozen immediately after feeding on individual birds. Table B. Host viremia, blood parasite status, and % mosquitoes infected. Table C. Published sources used to generate the host viremia:mosquito infection relationship for baby chickens and Culex pipiens mosquitoes. Table D. Method used to compute comparative host competence index values. Table E. Method used to compute comparative host competence index values. (DOCX) [file pntd.0010835.s001.docx]

**S1 - Supporting Information**

**S1**. Determining Host Viremia

The average volume of blood taken by *Culex pipiens* when feeding on a Common grackle was determined as follows. Three pools of five mosquitoes each were weighed on a microbalance, yielding an average weight of 1.80 + 0.14 micrograms per unfed mosquito. Mosquitoes were then allowed to feed on an anesthetized grackle. Immediately after feeding, individual engorged mosquitoes were aspirated into a cage, chilled at 4^o^ C for a few minutes, and placed on filter paper-in a glass dish on crushed ice. Chilling arrested physiological processes and mitigated weight loss due to post-feeding diuresis. Seven engorged mosquitoes were weighed individually, yielding an average weight of 5.19 + 0.41 milligrams. The weight of each blood meal was calculated as the difference between individual engorged weights minus the averaged unfed weight to yield an average of 3.39 + 0.41 ml of blood. This value was then divided by the specific gravity of adult chicken blood (i.e., 1.044 [1]) to yield an average blood meal volume of 3.25 microliters of blood.

For each infectious feed, two engorged mosquitoes were collected and triturated individually in 1 ml of media. Serial dilutions were prepared and assayed by plaque assay on Vero cells [2] to determine the quantity of virus present in each blood meal. Most blood meals (53 of 86) were assayed in duplicate on different dates to account for potential variation in plaque assays. The number of plaque-forming units (PFU) in a blood meal (*i.e*., PFU per 3.25 μl blood) was converted to PFU per ml of blood by multiplying by a factor of 10^2.5^ – that is, 3.25 μl multiplied by 10^2.5^ (*i.e*., 316.22) equals 1,028 μl, or 1.028 ml. In practice, we simply added 2.5 to the log_10_ PFU per bloodmeal values to convert to log_10_ PFU per ml. Values for log1_0_ PFU per ml were then averaged to estimate host viremia at the time of mosquito feeding.

A total of 41 viremias (23 grackle and 18 robin) were measured in this way, involving a total of 139 plaque assay titrations. Of these, 28 viremias (17 grackles and 10 robins; 100 plaque assay titrations total) had associated data on subsequent mosquito infection and thus comprise the host viremias used in further analysis for this report. For completeness, raw data on all 41 viremias are presented in Table A, below.

**Table A**: Host Viremias of grackles and robins as determined by plaque assay of engorged mosquitoes frozen immediately after feeding on individual birds.

| Bird Species & ID | Sex & weight (gm) | Date of Capture | Date of Feed | Day of Infection | Mosq No. | Plaque Assay Date | Log_10_ PFU per Blood Meal | Log_10_ PFU per ml Host Blood | Average Titer per ml Host Blood | Notes |
| --- | --- | --- | --- | --- | --- | --- | --- | --- | --- | --- |
| Grackle  1 | ♀  102 | 6/13/2011 | 4/4/2014 | 1 | 1 | 4/5/2014 | 3.3 | 5.8 | 6.17 |  |
|  |  |  |  |  | 2 | 4/8/2014 | 3.8 | 6.3 |  |  |
|  |  |  |  |  | 2 | 4/8/2014 | 3.9 | 6.4 |  |  |
|  |  |  | 4/5/2014 | 2 | 1 | 4/5/2014 | 3.8 | 6.3 | 6.33 |  |
|  |  |  |  |  | 1 | 4/5/2014 | 4.1 | 6.6 |  |  |
|  |  |  |  |  | 2 | 4/8/2014 | 3.5 | 6.0 |  |  |
|  |  |  |  |  | 2 | 4/8/2014 | 3.9 | 6.4 |  |  |
| Grackle  10 | ♀  108 | 5/5/2013 | 8/12/2013 | 1 | 1 | 8/19/2013 | 4.9 | 7.4 | 7.73 |  |
|  |  |  |  |  | 2 | 8/19/2013 | 5.1 | 7.6 |  |  |
|  |  |  |  |  | 1 | 9/3/2013 | 5.4 | 7.9 |  |  |
|  |  |  |  |  | 2 | 9/3/2013 | 5.5 | 8.0 |  |  |
|  |  |  | 8/13/2013 | 2 | 1 | 8/19/2013 | 5.3 | 7.8 | 8.00 |  |
|  |  |  |  |  | 2 | 8/19/2013 | 5.7 | 8.2 |  |  |
|  |  |  |  |  | 1 | 9/3/2013 | 5.4 | 7.9 |  |  |
|  |  |  |  |  | 2 | 9/3/2013 | 5.6 | 8.1 |  |  |
|  |  |  | 8/14/2013 | 3 | 1 | 8/19/2013 | 3.5 | 6.0 | 6.13 |  |
|  |  |  |  |  | 2 | 8/19/2013 | 3.7 | 6.2 |  |  |
|  |  |  |  |  | 1 | 9/3/2013 | 3.8 | 6.3 |  |  |
|  |  |  |  |  | 2 | 9/3/2013 | 3.5 | 6.0 |  |  |

**Table A**: Host Viremias (continued)

| Bird Species & ID | Sex & weight (gm) | Date of Capture | Date of Feed | Day of Infection | Mosq No. | Plaque Assay Date | Log_10_ PFU per Blood Meal | Log_10_ PFU per ml Host Blood | Average Titer per ml Host Blood | Notes |
| --- | --- | --- | --- | --- | --- | --- | --- | --- | --- | --- |
| Grackle  11 | ♂  99 | 4/9/2012 | 8/12/2013 | 1 | 1 | 8/19/2013 | 4.5 | 7.0 | 7.03 |  |
|  |  |  |  |  | 2 | 8/19/2013 | 4.3 | 6.8 |  |  |
|  |  |  |  |  | 1 | 9/3/2013 | 4.7 | 7.2 |  |  |
|  |  |  |  |  | 2 | 9/3/2013 | 4.6 | 7.1 |  |  |
|  |  |  | 8/13/2013 | 2 | 1 | 8/19/2013 | 4.2 | 6.7 | 6.68 |  |
|  |  |  |  |  | 2 | 8/19/2013 | 4.7 | 7.2 |  |  |
|  |  |  |  |  | 1 | 9/3/2013 | 4.0 | 6.5 |  |  |
|  |  |  |  |  | 2 | 9/3/2013 | 3.8 | 6.3 |  |  |
|  |  |  | 8/14/2013 | 3 | 1 | 8/19/2013 | 2.3 | 4.8 | 4.48 | Mosquito infection rates from this feed were not determined and thus this infectious feed was not part of the analysis. |
|  |  |  |  |  | 2 | 8/19/2013 | 2.1 | 4.6 |  |  |
|  |  |  |  |  | 1 | 9/3/2013 | 1.5 | 4.0 |  |  |
|  |  |  |  |  | 2 | 9/3/2013 | 2.0 | 4.5 |  |  |
| Grackle  23 | ♂  120 | 4/12/2012 | 8/12/2013 | 1 | 1 | 8/19/2013 | 3.9 | 6.4 | 6.68 |  |
|  |  |  |  |  | 2 | 8/19/2013 | 4.1 | 6.6 |  |  |
|  |  |  |  |  | 1 | 9/3/2013 | 4.1 | 6.6 |  |  |
|  |  |  |  |  | 2 | 9/3/2013 | 4.6 | 7.1 |  |  |
|  |  |  | 8/13/2013 | 2 | 1 | 8/19/2013 | 3.7 | 6.2 | 6.53 |  |
|  |  |  |  |  | 2 | 8/19/2013 | 4.2 | 6.7 |  |  |
|  |  |  |  |  | 1 | 9/3/2013 | 4.0 | 6.5 |  |  |
|  |  |  |  |  | 2 | 9/3/2013 | 4.2 | 6.7 |  |  |
|  |  |  | 8/14/2013 | 3 | 1 | 8/19/2013 | 1.0 | 2.5 | 2.50 | Mosquito infection rates from this feed were not determined and thus this infectious feed was not part of the analysis. |
|  |  |  |  |  | 2 | 9/3/2013 | 1.0 | 2.5 |  |  |

**Table A**: Host Viremias (continued)

| Bird Species & ID | Sex & weight (gm) | Date of Capture | Date of Feed | Day of Infection | Mosq No. | Plaque Assay Date | Log_10_ PFU per Blood Meal | Log_10_ PFU per ml Host Blood | Average Titer per ml Host Blood | Notes |
| --- | --- | --- | --- | --- | --- | --- | --- | --- | --- | --- |
| Grackle  29 | ♂  98 | 4/19/2012 | 8/12/2013 | 1 | 1 | 8/19/2013 | 3.6 | 6.1 | 6.35 |  |
|  |  |  |  |  | 2 | 8/19/2013 | 3.8 | 6.3 |  |  |
|  |  |  |  |  | 1 | 9/3/2013 | 3.9 | 6.4 |  |  |
|  |  |  |  |  | 2 | 9/3/2013 | 4.1 | 6.6 |  |  |
|  |  |  | 8/13/2013 | 2 | 1 | 8/19/2013 | 3.2 | 5.7 | 5.95 |  |
|  |  |  |  |  | 2 | 8/19/2013 | 3.6 | 6.1 |  |  |
|  |  |  |  |  | 1 | 9/3/2013 | 3.3 | 5.8 |  |  |
|  |  |  |  |  | 2 | 9/3/2013 | 3.7 | 6.2 |  |  |
|  |  |  | 8/14/2013 | 3 | 1 | 8/19/2013 | 0 | 2.5 | 3.00 | Mosquito infection rates from this feed were not determined and thus this infectious feed was not part of the analysis. |
|  |  |  |  |  | 2 | 9/3/2013 | 1.0 | 3.5 |  |  |
| Grackle  39 | ♀  95 | 5/12/2013 | 8/12/2013 | 1 | 1 | 8/19/2013 | 4.9 | 7.4 | 7.53 |  |
|  |  |  |  |  | 2 | 8/19/2013 | 5.2 | 7.7 |  |  |
|  |  |  |  |  | 1 | 9/3/2013 | 4.9 | 7.4 |  |  |
|  |  |  |  |  | 2 | 9/3/2013 | 5.1 | 7.6 |  |  |
|  |  |  | 8/13/2013 | 2 | 1 | 8/19/2013 | 5.7 | 8.2 | 8.28 |  |
|  |  |  |  |  | 2 | 8/19/2013 | 5.7 | 8.2 |  |  |
|  |  |  |  |  | 1 | 9/3/2013 | 5.9 | 8.4 |  |  |
|  |  |  |  |  | 2 | 9/3/2013 | 5.8 | 8.3 |  |  |
|  |  |  | 8/14/2013 | 3 | 1 | 8/19/2013 | 5.5 | 8.0 | 7.90 |  |
|  |  |  |  |  | 2 | 8/19/2013 | 5.3 | 7.8 |  |  |
|  |  |  |  |  | 1 | 9/3/2013 | 5.4 | 7.9 |  |  |
|  |  |  |  |  | 2 | 9/3/2013 | 5.4 | 7.9 |  |  |

**Table A**: Host Viremias (continued)

| Bird Species & ID | Sex & weight (gm) | Date of Capture | Date of Feed | Day of Infection | Mosq No. | Plaque Assay Date | Log_10_ PFU per Blood Meal | Log_10_ PFU per ml Host Blood | Average Titer per ml Host Blood | Notes |
| --- | --- | --- | --- | --- | --- | --- | --- | --- | --- | --- |
| Grackle  42 | ♂  104 | 4/23/2012 | 8/12/2013 | 1 | 1 | 8/19/2013 | 6.1 | 8.6 | 8.70 | Grackle 42 was both microfilaremic and highly viremic which produced significant enhancement of West Nile virus infection and dissemination.  (Vaughan et al. 2021)  Therefore, Grackle 42 was excluded from this analysis. |
|  |  |  |  |  | 2 | 8/19/2013 | 6.0 | 8.5 |  |  |
|  |  |  |  |  | 1 | 9/3/2013 | 6.6 | 9.1 |  |  |
|  |  |  |  |  | 2 | 9/3/2013 | 6.1 | 8.6 |  |  |
|  |  |  | 8/13/2013 | 2 | 1 | 8/19/2013 | 6.3 | 8.8 | 9.13 |  |
|  |  |  |  |  | 2 | 8/19/2013 | 6.4 | 8.9 |  |  |
|  |  |  |  |  | 1 | 9/3/2013 | 7.1 | 9.6 |  |  |
|  |  |  |  |  | 2 | 9/3/2013 | 6.7 | 9.2 |  |  |
| Grackle  53 | ♂  103 | 5/22/2013 | 4/4/2014 | 1 | 1 | 4/5/2014 | 4.0 | 6.5 | 6.38 |  |
|  |  |  |  |  | 1 | 4/5/2014 | 4.1 | 6.6 |  |  |
|  |  |  |  |  | 2 | 4/8/2014 | 3.5 | 6.0 |  |  |
|  |  |  |  |  | 2 | 4/8/2014 | 3.9 | 6.4 |  |  |
|  |  |  | 4/5/2014 | 2 | 1 | 4/5/2014 | 3.7 | 6.2 | 6.33 |  |
|  |  |  |  |  | 1 | 4/5/2014 | 4.2 | 6.7 |  |  |
|  |  |  |  |  | 2 | 4/8/2014 | 3.6 | 6.1 |  |  |
|  |  |  |  |  | 2 | 4/8/2014 | 3.8 | 6.3 |  |  |
| Grackle  152 | ♂  124 | 6/11/2011 | 4/4/2014 | 1 | 1 | 4/5/2014 | 3.9 | 6.4 | 6.40 | Mosquito infection rates from this feed were not determined and thus this infectious feed was not part of the analysis. |
|  |  |  |  |  | 1 | 4/5/2014 | 4.1 | 6.6 |  |  |
|  |  |  |  |  | 2 | 4/8/2014 | 3.6 | 6.1 |  |  |
|  |  |  |  |  | 2 | 4/8/2014 | 4.0 | 6.5 |  |  |
|  |  |  | 4/5/2014 | 2 | 1 | 4/5/2014 | 3.0 | 5.5 | 5.65 |  |
|  |  |  |  |  | 1 | 4/5/2014 | 3.3 | 5.8 |  |  |
|  |  |  |  |  | 2 | 4/8/2014 | 3.4 | 5.9 |  |  |
|  |  |  |  |  | 2 | 4/8/2014 | 2.9 | 5.4 |  |  |

**Table A**: Host Viremias (continued)

| Bird Species & ID | Sex & weight (gm) | Date of Capture | Date of Feed | Day of Infection | Mosq No. | Plaque Assay Date | Log_10_ PFU per Blood Meal | Log_10_ PFU per ml Host Blood | Average Titer per ml Host Blood | Notes |
| --- | --- | --- | --- | --- | --- | --- | --- | --- | --- | --- |
| Robin  3 | n.d.  76 | 4/18/2014 | 8/13/2014 | 1 | 1 | 8/15/2014 | 3.9 | 6.4 | 6.67 |  |
|  |  |  |  |  | 1 | 8/18/2014 | 4.3 | 6.8 |  |  |
|  |  |  |  |  | 2 | 8/18/2014 | 4.3 | 6.8 |  |  |
|  |  |  | 8/14/2014 | 2 | 1 | 8/15/2014 | 4.0 | 6.5 | 6.80 | Mosquito infection rates from this feed were not determined and thus this infectious feed was not part of the analysis. |
|  |  |  |  |  | 2 | 8/18/2014 | 4.6 | 7.1 |  |  |
|  |  |  |  |  | 1 | 8/18/2014 | 4.3 | 6.8 |  |  |
|  |  |  | 8/15/2014 | 3 | 2 | 8/18/2014 | 1.3 | 3.8 | 4.40 | Mosquito infection rates from this feed were not determined and thus this infectious feed was not part of the analysis. |
|  |  |  |  |  | 1 | 8/18/2014 | 0 | 0 |  |  |
| Robin  52 | n.d.  83 | 5/20/2014 | 8/13/2014 | 1 | 1 | 8/15/2014 | 4.5 | 7 | 6.90 |  |
|  |  |  |  |  | 1 | 8/18/2014 | 4.4 | 6.9 |  |  |
|  |  |  |  |  | 2 | 8/18/2014 | 4.3 | 6.8 |  |  |
|  |  |  | 8/14/2014 | 2 | 1 | 8/15/2014 | 4.7 | 7.2 | 7.17 |  |
|  |  |  |  |  | 1 | 8/18/2014 | 4.7 | 7.2 |  |  |
|  |  |  |  |  | 2 | 8/18/2014 | 4.6 | 7.1 |  |  |
|  |  |  | 8/15/2014 | 3 | 1 | 8/15/2014 | 2.9 | 5.4 | 5.20 | Mosquito infection rates from this feed were not determined and thus this infectious feed was not part of the analysis. |
|  |  |  |  |  | 2 | 8/18/2014 | 2.5 | 5.0 |  |  |
|  |  |  |  |  | 3 | 8/18/2014 | 2.7 | 5.2 |  |  |

**Table A**: Host Viremias (continued)

| Bird Species & ID | Sex & weight (gm) | Date of Capture | Date of Feed | Day of Infection | Mosq No. | Plaque Assay Date | Log_10_ PFU per Blood Meal | Log_10_ PFU per ml Host Blood | Average Titer per ml Host Blood | Notes |
| --- | --- | --- | --- | --- | --- | --- | --- | --- | --- | --- |
| Robin  55 | n.d.  83 | 5/21/2014 | 8/13/2014 | 1 | 1 | 8/15/2014 | 4.6 | 7.1 | 7.37 |  |
|  |  |  |  |  | 1 | 8/18/2014 | 5.0 | 7.5 |  |  |
|  |  |  |  |  | 2 | 8/18/2014 | 5.0 | 7.5 |  |  |
|  |  |  | 8/14/2014 | 2 | 1 | 8/15/2014 | 4.4 | 6.9 | 6.90 |  |
|  |  |  |  |  | 1 | 8/18/2014 | 4.4 | 6.9 |  |  |
|  |  |  |  |  | 2 | 8/18/2014 | 4.4 | 6.9 |  |  |
|  |  |  | 8/15/2014 | 3 | 1 | 8/15/2014 | <2 |  | 4.10 | Mosquito infection rates from this feed were not determined and thus this infectious feed was not part of the analysis. |
|  |  |  |  |  | 2 | 8/18/2014 | 1.6 | 4.1 |  |  |
| Robin  67 | n.d.  97 | 5/28/2014 | 8/13/2014 | 1 | 1 | 8/15/2014 | 4.1 | 6.6 | 6.53 |  |
|  |  |  |  |  | 1 | 8/18/2014 | 3.8 | 6.3 |  |  |
|  |  |  |  |  | 2 | 8/18/2014 | 4.2 | 6.7 |  |  |
|  |  |  | 8/14/2014 | 2 | 1 | 8/15/2014 | 2.0 | 4.5 | 4.50 |  |
|  |  |  |  |  | 1 | 8/18/2014 | 2.5 | 5.0 |  |  |
|  |  |  |  |  | 2 | 8/18/2014 | 1.5 | 4 |  |  |
|  |  |  | 8/15/2014 | 3 | 1 | 8/15/2014 | 0 | 0 | 0 | Mosquito infection rates from this feed were not determined and thus this infectious feed was not part of the analysis. |
|  |  |  |  |  | 2 | 8/18/2014 | 0 | 0 |  |  |
|  |  |  |  |  | 3 | 8/18/2014 | 0 | 0 |  |  |

**Table A**: Host Viremias (continued)

| Bird Species & ID | Sex & weight (gm) | Date of Capture | Date of Feed | Day of Infection | Mosq No. | Plaque Assay Date | Log_10_ PFU per Blood Meal | Log_10_ PFU per ml Host Blood | Average Titer per ml Host Blood | Notes |
| --- | --- | --- | --- | --- | --- | --- | --- | --- | --- | --- |
| Robin  89 | n.d.  81 | 6/18/2014 | 8/13/2014 | 1 | 1 | 8/15/2014 | 4.6 | 7.1 | 7.33 |  |
|  |  |  |  |  | 1 | 8/18/2014 | 5.0 | 7.5 |  |  |
|  |  |  |  |  | 2 | 8/18/2014 | 4.9 | 7.4 |  |  |
|  |  |  | 8/14/2014 | 2 | 1 | 8/15/2014 | 4 | 6.5 | 6.70 |  |
|  |  |  |  |  | 1 | 8/18/2014 | 4.4 | 6.9 |  |  |
|  |  |  |  |  | 2 | 8/18/2014 | 4.2 | 6.7 |  |  |
|  |  |  | 8/15/2014 | 3 | 1 | 8/15/2014 | <2.0 |  | 4.10 | Mosquito infection rates from this feed were not determined and thus this infectious feed was not part of the analysis. |
|  |  |  |  |  | 2 | 8/18/2014 | <2.0 |  |  |  |
|  |  |  |  |  | 3 | 8/18/2014 | 1.6 | 4.1 |  |  |
| Robin  108 | n.d.  82 | 6/25/2014 | 8/13/2014 | 1 | 1 | 8/15/2014 | 4.3 | 6.8 | 7.00 |  |
|  |  |  |  |  | 1 | 8/18/2014 | 4.9 | 7.4 |  |  |
|  |  |  |  |  | 2 | 8/18/2014 | 4.3 | 6.8 |  |  |
|  |  |  | 8/14/2014 | 2 | 1 | 8/15/2014 | 4.6 | 7.1 | 7.30 |  |
|  |  |  |  |  | 1 | 8/18/2014 | 4.9 | 7.4 |  |  |
|  |  |  |  |  | 2 | 8/18/2014 | 4.9 | 7.4 |  |  |
|  |  |  | 8/15/2014 | 3 | 1 | 8/15/2014 | <2.0 |  | 4.35 | Mosquito infection rates from this feed were not determined and thus this infectious feed was not part of the analysis. |
|  |  |  |  |  | 2 | 8/18/2014 | 1.8 | 4.3 |  |  |
|  |  |  |  |  | 3 | 8/18/2014 | 1.9 | 4.4 |  |  |

**Table** **B** – West Nile virus infection prevalence in *Culex pipiens* mosquitoes after feeding on viremic birds with or without concurrent blood parasite infection.

| **Bird Species** | **Bird ID** | **WNV Virus Titer** | **Mosquito Strain** | **Percent Infected** | **Number Sampled** | **----------- Blood Parasite Status -----------** | | |
| --- | --- | --- | --- | --- | --- | --- | --- | --- |
|  |  |  |  |  |  | **Microfilariae** | ***Plasmodium*** | **Trypansomes** |
| Grackle | 152 | 5.65 | Area B | 40.0% | 20 | positive | negative | positive |
|  |  |  | Rutgers | 27.3% | 11 |  |  |  |
| Grackle | 29 | 5.95 | Area B | 10.0% | 10 | positive | negative | negative |
| Grackle | 10 | 6.13 | Area B | 5.0% | 20 | negative | negative | negative |
|  |  |  | Rutgers | 0.0% | 20 |  |  |  |
| Grackle | 1 | 6.17 | Area B | 53.3% | 15 | negative | negative | negative |
|  |  |  | Rutgers | 8.7% | 23 |  |  |  |
|  |  | 6.33 | Area B | 50.0% | 40 |  |  |  |
|  |  |  | Rutgers | 34.6% | 26 |  |  |  |
| Grackle | 53 | 6.33 | Area B | 56.6% | 53 | positive | negative | negative |
|  |  |  | Rutgers | 29.7% | 37 |  |  |  |
| Grackle | 29 | 6.35 | Area B | 45.7% | 35 | positive | negative | negative |
| Grackle | 53 | 6.38 | Area B | 49.1% | 53 | positive | negative | negative |
|  |  |  | Rutgers | 21.1% | 19 |  |  |  |
| Grackle | 23 | 6.53 | Area B | 31.7% | 60 | positive | negative | negative |
| Grackle | 11 | 6.68 | Area B | 24.0% | 50 | negative | negative | negative |
| Grackle | 23 | 6.68 | Area B | 71.7% | 53 | positive | negative | negative |
| Grackle | 11 | 7.03 | Rutgers | 4.0% | 25 | negative | negative | negative |
| Grackle | 39 | 7.53 | Rutgers | 51.4% | 37 | negative | negative | negative |
| Grackle | 10 | 7.73 | Rutgers | 48.0% | 50 | negative | negative | negative |
| Grackle | 39 | 7.90 | Rutgers | 68.1% | 47 | negative | negative | negative |
| Grackle | 10 | 8.00 | Area B | 63.3% | 60 | negative | negative | negative |
| Grackle | 39 | 8.28 | Rutgers | 65.1% | 63 | negative | negative | negative |
| Grackle | 42 * | 8.70 | Area B | 100.0% | 50 | positive | negative | negative |
|  |  | 9.13 | Rutgers | 95.9% | 49 |  |  |  |
| Robin | 67 | 4.50 | Area B | 7.7% | 13 | positive | positive | positive |
|  |  |  | Rutgers | 10.0% | 10 |  |  |  |
|  |  | 6.53 | Area B | 100.0% | 13 |  |  |  |
|  |  |  | Rutgers | 90.3% | 31 |  |  |  |
| Robin | 3 | 6.67 | Area B | 100.0% | 19 | positive | positive | positive |
|  |  |  | Rutgers | 85.7% | 14 |  |  |  |
| Robin | 89 | 6.70 | Area B | 100.0% | 34 | negative | negative | negative |
|  |  |  | Rutgers | 73.5% | 34 |  |  |  |
| Robin | 52 | 6.90 | Rutgers | 90.0% | 10 | negative | positive | negative |
| Robin | 108 | 7.00 | Area B | 100.0% | 11 | positive | negative | positive |
|  |  |  | Rutgers | 100.0% | 23 |  |  |  |
| Robin | 52 | 7.17 | Area B | 100.0% | 20 | negative | positive | negative |
|  |  |  | Rutgers | 100.0% | 20 |  |  |  |
| Robin | 108 | 7.30 | Area B | 90.9% | 11 | positive | negative | positive |
|  |  |  | Rutgers ** | 71.4% | 21 |  |  |  |
| Robin | 89 | 7.33 | Area B | 89.3% | 28 | negative | negative | negative |
|  |  |  | Rutgers | 91.4% | 35 |  |  |  |
| Robin | 55 | 7.37 | Area B | 100.0% | 38 | negative | positive | negative |
|  |  |  | Rutgers | 100.0% | 14 |  |  |  |

* excluded from analysis due to the occurrence of microfilarial enhancement (Vaughan *et al*. 2021 [ref. 3])

** excluded from analysis because it was determined to be a statistical outlier.

**S1**. Determining species-specific reservoir competence index values; refer to Tables C, D & E.

1. Define the VIREMIA – INFECTIOUSNESS RELATIONSHIP – compared 2 methods

A) Measure host viremia and extrapolate infectiousness to mosquitoes by using the regression equation generated from mosquito feedings on viremic baby chickens. (see Table C and Fig A, below)

B) Measure both viremia and mosquito infectiousness experimentally for different bird species.

2. For a set of viremic birds, measure their individual daily viremias.

3. From the individual daily viremia, use the regression equation or empirical data on mosquito infection prevalence to:

- Calculate the average proportion of mosquitoes infected by the viremic birds on DAY 1

- Calculate the average proportion of mosquitoes infected by the viremic birds on Day 2

- Calculate the average proportion of mosquitoes infected by the viremic birds on Day 3

….. and so forth, until the viremia wanes and no more mosquitoes are infected (or the bird dies!)

Then sum the average infectiousness for Days 1, 2, and 3 to yield the Reservoir Competence, **‘*C’***

**Table C** – Data and their published sources which were used to generate the regression equation that describes the host viremia (log_10_ PFU/mL) : mosquito infectiousness (%) relationship when *Culex pipiens* mosquitoes were fed directly on viremic baby chickens infected with the NY-1999 (crow) [4, 5] / Crow 397-99 [6] strains of West Nile virus.

| Host Viremia expressed as cell culture infective dose (CID)_50_ per mL | Host Viremia expressed as log_10_ plaque-forming units (PFU) per mL * | Percent Infected | N | References |
| --- | --- | --- | --- | --- |
| 5.0 | 4.501 | 2.9% | 70 | 4, 5 |
| 5.5 | 4.968 | 15.4% | 65 | 4, 5 |
|  | 5.2 | 17.4% | 46 | 6 |
| 6 | 5.436 | 50.0% | 24 | 4 |
| 6.5 | 5.903 | 68% | 50 | 4, 5 |
| 7.0 | 6.371 | 97.0% | 67 | 4 |
| 7.5 | 6.838 | 96.4% | 83 | 4, 5 |
|  | 7.2 | 80.7% | 83 | 6 |
| 8.0 | 7.306 | 97.2% | 72 | 4, 5 |
| 8.5 | 7.773 | 98% | 52 | 4, 5 |

Note: Titers expressed as CID50 per mL were converted to log_10_ PFU per mL using the equation: 0.935 (CID50/mL) – 0.174 [see Reference 4].

Equation: Y=0.315x-1.3143 R^2^ = 0.8449

Data are graphed and presented in Fig A below.

**Fig A**. Regression line showing host viremia (log_10_ PFU/mL) : mosquito infectiousness (%) relationship when *Culex pipiens* mosquitoes were fed directly on viremic baby chickens infected with the NY-1999 (crow) [4, 5] / Crow 397-99 [6] strains of West Nile virus.

**Table D** – Comparing mosquito infection prevalence (both mosquito strains combined) after feeding on West Nile virus infected grackles and robins, as determined theoretically by using regression equation generated by published data with viremic baby chickens (see Table C, above) versus actual experimental infections with grackles and robins. Data are sorted by bird species, day of viremia, and viremia. Columns and rows are labeled in typical spreadsheet format.

|  | **A** | **B** | **C** | **D** | **E** | **F** |
| --- | --- | --- | --- | --- | --- | --- |
| **1** | Bird Species | Bird ID | Viremia | Day of Viremia | Estimated % mosquito infected extrapolated from baby chicken regression equation | Actual % mosquito infected (both mosquito strains combined) |
| **2** | Grackle | 10 | 7.7 | 1 | 100% | 48% |
| **3** | Grackle | 39 | 7.5 | 1 | 105% | 51% |
| **4** | Grackle | 11 | 7.0 | 1 | 89% | 4% |
| **5** | Grackle | 23 | 6.7 | 1 | 80% | 72% |
| **6** | Grackle | 29 | 6.4 | 1 | 70% | 46% |
| **7** | Grackle | 53 | 6.4 | 1 | 70% | 42% |
| **8** | Grackle | 1 | 6.2 | 1 | 64% | 26% |
| **9** | Grackle | 152 | 5.7 | 1 | 48% | 35% |
| **10** | Grackle | 39 | 8.3 | 2 | 100% | 65% |
| **11** | Grackle | 10 | 8.0 | 2 | 100% | 63% |
| **12** | Grackle | 11 | 6.7 | 2 | 80% | 24% |
| **13** | Grackle | 23 | 6.5 | 2 | 73% | 32% |
| **14** | Grackle | 53 | 6.3 | 2 | 67% | 47% |
| **15** | Grackle | 1 | 6.3 | 2 | 67% | 44% |
| **16** | Grackle | 29 | 6.0 | 2 | 58% | 10% |
| **17** | Grackle | 39 | 7.9 | 3 | 100% | 68% |
| **18** | Grackle | 10 | 6.1 | 3 | 61% | 3% |
| **19** | Robin | 55 | 7.4 | 1 | 101% | 100% |
| **20** | Robin | 89 | 7.3 | 1 | 100% | 90% |
| **21** | Robin | 108 | 7.0 | 1 | 89% | 100% |
| **22** | Robin | 52 | 6.9 | 1 | 86% | 90% |
| **23** | Robin | 3 | 6.7 | 1 | 80% | 94% |
| **24** | Robin | 67 | 6.5 | 1 | 74% | 93% |
| **25** | Robin | 108 | 7.3 | 2 | 99% | 91% |
| **26** | Robin | 52 | 7.2 | 2 | 94% | 100% |
| **27** | Robin | 89 | 6.7 | 2 | 80% | 87% |
| **28** | Robin | 67 | 4.5 | 2 | 0% | 9% |

**Table E**. Calculations used to determine host competence index values for grackles and robins when applying regression equation from baby chicken infections (column K) versus using actual experimental result from grackle and robin infections (column L). Columns and rows are labeled in typical spreadsheet format. Note: formulas in italics represent Excel script. Some of the formulas refer to column/row locations in Table D above.

|  | **H** | **I** | **J** | **K** | | **L** | |
| --- | --- | --- | --- | --- | --- | --- | --- |
| **1** | **Bird Species** | **DAILY INFECTIOUSNESS ("*I*")** | **N** | **Derived from Baby Chicken Equation** | | **Derived from Actual Data** | |
| **2** | **Grackle** | Average Infectiousness DAY 1 | 8 | 0.78 | *=average(E2:E9)* | 0.41 | *=average(F2:F9)* |
| **3** | **Grackle** | Average Infectiousness DAY 2 | 7 | 0.78 | *=average(E10:E16)* | 0.41 | *=average(F10:F16)* |
| **4** | **Grackle** | Average Infectiousness DAY 3 | 2 | 0.80 | *=average(E17:E18)* | 0.35 | *=average(F17:F18)* |
| **5** | SUM | | | **2.36** | *=sum(K2:K4)* | **1.17** | *=sum(L2:L4)* |
| **6** | Mean | | | 0.78 | *=average(E2:E18)* | 1.20 | *=average(F2:F18)* |
| **7** | Standard Deviation | | | 0.176 | *=stdev(E2:E18)* | 0.215 | *=stdev(F2:F18)* |
| **8** | Sample Size | | | 17 | *=count(E2:E18)* | 17 | *=count(F2:F18)* |
| **9** | Confidence Coefficient | | | 1.96 | *1.96* | 1.96 | *1.96* |
| **10** | Margin of Error | | | 0.084 | *=(K9*K7/K8^0.5)* | 0.102 | *=(L9*L7/L8^0.5)* |
| **11** | Upper Bound | | | 2.45 | *=K5+K10* | 1.27 | *=L5+L10* |
| **12** | Lower Bound | | | 2.28 | *=K5-K10* | 1.06 | *=L5-L10* |
| **13** |  |  |  |  |  |  |  |
| **14** | **Robin** | Average Infectiousness DAY 1 | 6 | 0.88 | *=average(E19:E24)* | 0.95 | *=average(F19:F24)* |
| **15** | **Robin** | Average Infectiousness DAY 2 | 4 | 0.68 | *=average(E25:E28)* | 0.72 | *=average(F25:F28)* |
| **16** | SUM | | | **1.56** | *=sum(K14:K15)* | **1.66** | *=sum(L14:L15)* |
| **17** | Mean | | | 0.78 | *=average(K14:K15)* | 0.83 | *=average(L14:L15)* |
| **18** | Standard Deviation | | | 0.296 | *=stdev(K14:K15)* | 0.274 | *=stdev(L14:L15)* |
| **19** | Sample Size | | | 10 | *=count(K14:K15)* | 10 | *=count(L14:L15)* |
| **20** | Confidence Coefficient | | | 1.96 | *1.96* | 1.96 | *1.96* |
| **21** | Margin of Error | | | 0.184 | *=(K20*K18/K19^0.5)* | 0.169 | *=(L20*L18/L19^0.5)* |
| **22** | Upper Bound | | | 1.75 | *K16+K21* | 1.83 | *L16+L21* |
| **23** | Lower Bound | | | 1.38 | *K16-K21* | 1.49 | *L16-L21* |

**References**

1. Medway W, Kare MR. Blood and plasma volume, hematocrit, blood specific gravity and serum protein electrophoresis of the chicken. Poultry Sci. 1959;38: 624-631.
2. Turell MJ, O’GuinnM, Oliver J. Potential for New York mosquitoes to transmit West Nile virus. Am J Trop Med Hyg. 2000;62:413-414.
3. Tiawsirisup S, Platt KB, Evans RB, Rowley WA. Susceptibility of *Ochlerotatus trivittatus* (Coq.), *Aedes albopictus* (Skuse), and *Culex pipiens* (L.) to West Nile virus infection. Vector Borne Zoonotic Dis. 2004;4: 190-197. doi: 10.1089/vbz.2004.4.190.
4. Vaughan JA, Hinson J, Andrews ES, Turell MJ. Pre-existing microfilarial infections of American Robins (Passeriformes: Turdidae) and Common Grackles (Passeriformes: Icteridae) have limited impact on enhancing dissemination of West Nile virus in *Culex pipiens* mosquitoes (Diptera: Culicidae). J Med Entomol. 2021;58(3):1389-1397. doi: 10.1093/jme/tjaa261
5. Tiawsirisup S, Platt KB, Evans RB, Rowley WA. A comparison of West Nile Virus transmission by *Ochlerotatus trivittatus* (COQ.), *Culex pipie*ns (L.), and *Aedes albopictus* (Skuse). Vector Borne Zoonotic Dis. 2005;5: 40-47. doi: 10.1089/vbz.2005.5.40.
6. Turell MJ, O'Guinn M, Oliver J. Potential for New York mosquitoes to transmit West Nile virus. Am J Trop Med Hyg. 2000;62: 413-414. doi: 10.4269/ajtmh.2000.62.413.
