# Supplementary material for: Bird species define the relationship between West Nile viremia and infectiousness to Culex pipiens mosquitoes
Source: PLoS Negl Trop Dis. 2022 Oct 6;16(10):e0010835. doi: 10.1371/journal.pntd.0010835 (PMC9578590; doi:10.1371/journal.pntd.0010835)
Supplement: Attachment — Submitted filename: Vaughan ms PLOS NTD 2022 comments for authors.pdf [file pntd.0010835.s003.pdf]

General comments

This ms examines an important question: to what extent can measures of viremia in a host be used to infer the infectiousness to biting vectors, without feeding vectors on hosts directly? Previous work in multiple systems has quantified the viremia-infectiousness relationship using artificial blood feeding systems and used this to translate viremias into infectiousness. The study here examines the fraction of mosquitoes infected after feeding on two species of birds – American robin and common grackle (and comparing data to studies of baby chickens). They find that the fraction of mosquitoes infected after feeding on a host with a given viremia is very similar for chickens and robins, but substantially lower for grackles. They conclude that host infectiousness cannot be inferred without measuring mosquito feeding on hosts.

While this is an important study, there are many substantial issues with the study that greatly limit the applicability of the conclusions that can be drawn. Some of the major issues are:

-The study only discusses mosquito infection, not disseminated infection or transmission. Infection is the least epidemiologically important metric of vector competence and one that often gives very different results than disseminated infection or transmission. As I was preparing this review, I downloaded the supplemental material and discovered that disseminated infections were measured for a subset of the feedings. The disseminated infections data produce results that are completely different from the infection data – they show no statistical evidence of differences among birds in the fraction of mosquitoes with disseminated infections given the same viremia. See below for analysis of these data and a better analysis of the infection data.

- The study examines 2 wild bird species: robins and grackles. While the data are clear in showing that grackles infect a smaller fraction of mosquitoes than robins at similar levels of viremia, whether this is a substantial issue among other species is unclear. If grackles are simply anomalous and most hosts are similar and can be described using viremia patterns either in artificial systems or in chickens then the results found here are of minor importance.

- The study is done with highly inbred lines of mosquitoes. Whether similar results would be found with wild mosquitoes is totally unknown.

- The presentation of raw data and the statistical analysis are problematic. The viremia data aren't shown, in part because they have been plotted in another previous paper. They could easily be included as a supplemental table. The supplemental table that is included doesn't include the full viremia profiles that are used in the paper to estimate the host competence index.

- The link to the published literature is very poor. See below for details.

- The statistical analysis is problematic (it doesn't account for repeated feedings on the same birds which are pseudoreplicates), and a correct analysis is far weaker (and technically doesn't support the main conclusion). It suggests that most of the pattern seen is not due to differences among species, but due to differences among individual birds. See below for details.

In summary, this study claims that measures of viremia cannot be used to infer infectiousness to vectors. In actuality, this study shows that there might be some variation among species in the relationship between viremia and the fraction of mosquitoes infected (but the data do not provide

support to claim that the relationship between viremia and the fraction of mosquitoes with disseminated infections differs among species). More broadly, the fraction of vectors that will become infectious and transmit a pathogen after feeding on a host species depends on many things, in rough order of importance:

- Days post-feeding
- Variation among individuals
- Species average viremia
- Temperature post feeding
- Non-viremia factors that influence vector infection and viral dissemination

Thus, the strong conclusions of the paper are not supported by the data presented. The data in the paper (especially including the supplemental material on disseminated infections) could be used to support the much weaker claims that:

- The fraction of mosquitoes infected after feeding on an infected host may depend, in part, on non-viremic differences among species, but these differences are likely smaller than differences among species and individuals in viremia, and other variables include time since feeding and temperature, as well as among mosquito populations.

More importantly, there was no evidence for difference among birds in the fraction of mosquitoes with disseminated infections, but there was strong evidence that viremia was a significant predictor of disseminated infections. These results support the previous research that used viremia to predict the fraction of mosquitoes that would go on to transmit West Nile virus.

Specific comments

L36 Why “peak viremia”? Do you mean “viremia”? In the abstract you don’t give any reason for the infectiousness to be different for different birds. Is this possibly related to filarial worms or other things?

L46-63 There isn’t a single reference in the entire first paragraph. It’s also a very basic first paragraph that belongs in an introductory textbook, not a primarily literature paper. I’d strongly suggest deleting almost the entire paragraph and starting the paper off with a statement of the research gap that the paper is filling by introducing the reader to the vast vast literature on this topic. For every relevant sentence in this paragraph there are literally dozens of citations that would be useful to give the reader to introduce them to the previous science on this topic.

L56-7 This text makes it seem like vectors either transmit or don’t when, in fact, it is a continuous trait with most (but not all) species being able to transmit a little with high enough viremia and the right temperature and time. These species are very weakly competent vectors. Others are moderately competent and some are, relatively speaking, highly competent. But I’ve never seen any data showing any species that is 100% competent in that it always transmits after feeding on low viremias regardless of the temperature post feeding or the time post feeding. You are doing the reader a great disservice explaining this basic concept this way. See comment above – I’d delete almost this entire paragraph anyway.

L64 The claim of “most important” isn’t supported by data or citations here. One could easily refute this argument by showing that transmission following infection is zero at low temperatures. Remove this

unsupportable claim and add several references to give the reader a way to see previous work on this topic.

L68-70 Like the description above of "vectors" this categorization of hosts and "amplifying" or "dead-end" is again an oversimplification of a continuous trait into two categories that badly misleads the reader. Are European starlings "amplifying hosts"? Komar et al 2003 EID showed that they have viremias that are barely infectious and thus would rarely lead to mosquitoes that would transmit, but a very tiny fraction of mosquitoes would, if they fed on them on their day of peak viremia and were held at the right temperature for the right amount of time. Similarly, Rock doves were assigned a host competence value of 0 by Komar et al 2003 EID suggesting they were dead end hosts. Later work showed that with a larger sample of birds that were sampled in the field, some were weakly competent. If you are going to explain this basic concept, do so correctly and indicate that host competence is a continuous trait with different fraction of mosquitoes becoming infected and going on to transmit and that this depends not just on host viremia but also temperature, the day they are tested, whether they were food stressed as larvae, etc.

L68-70 There is huge variation among birds in viremias, so a single bird species doesn't have a single competence value. Some individual birds within a species are highly infectious whereas others are quite poor.

L75 This is the first citation in the paper. By this time you should have cited 10-20 other papers.

L121 The year for Ref 10 is incorrect. It is a 2005 paper, not 2004.

L122-127 I went back and read Ref 10 to examine this case and you've neglected to mention that the populations of mosquitoes feeding on the finch and the chicken are from different places in Kern county so it's not surprising at all that the fraction infected differed. Work by both Laura Kramer's lab (doi:10.4269/ajtmh.2010.10-0005) and Bill Reisen's group (https://doi.org/10.1093/jmedent/45.6.1126) showed that vector competence for a single species of mosquito can vary substantially over time and over short distances. In fact, none of the feedings of both chicks and finches in ref 10 used mosquitoes from the same population of mosquitoes so this study cannot be used to make claims about differences among species in infectivity to mosquitoes with the same viremia.

L144-145 24 hr is an extremely short acclimatization period. Most studies with wild birds use 2 weeks!

L156-8 These are highly inbred lines of mosquitoes. It's not clear to me that the results from these mosquitoes tell us much about how wild mosquitoes would perform in the same experiments.

L173 Why did you only test for infection? This is the least relevant of measures of vector competence. Salivary secretions would be most relevant but disseminated infections would at least demonstrate escape from the midgut. Analysis of the disseminated infection data in the supplemental material produce very different results (see below for analysis).

L165-171 What temperature were mosquitoes held at?

L179 Why not simply fit all the data to a GLM with a binomial distribution with species and viremia and species*viremia as predictors? This would answer the question in a much simpler way. In addition, since there are repeated feedings on the same birds the analysis must account for this and is easiest done with a mixed effects model (e.g. glmer). See below for this analysis.

L187 It's not clear how the statistics were done here, but a simple comparison suggests something is badly wrong. If the two slopes are 0.25 and 0.58 and the pooled SE of these values is ~0.09, then the t-value should be (0.58-0.25)/0.09 = 3.66. Given the very large difference in slopes relative to the SEs of the slopes it is definitely NOT valid to combine data from the two strains of mosquitoes. What strains of mosquitoes were fed on the robins?

L197-8 Which viremia are you using? An average? If the relationship isn't linear, then an average isn't appropriate. Further, what do you consider an infectious viremia? One that results in 1% of mosquitoes becoming infected? With the sample sizes you've used, you can't exclude low fractions of mosquitoes being infected.

L202 What is the IACUC protocol number covering this research?

L211 You absolutely must show the viremias in this paper since it the whole focus of the study.

L222 This isn't justified. See comments above.

L225-234 The analysis of this data is very messy and problematic. Instead, as suggested above, the authors should do a single GLM with the whole dataset with a binomial distribution and predictors for bird species, mosquito strain, viremia and interactions which will avoid arbitrary post-hoc combining of data. See below for this analysis that also includes random effects to account for repeatedly feeding on the same bird.

L260 Why are the "days of infectiousness" so low? The data in Komar et al 2003 EID suggest that both robins and grackles are partly infectious for at least 4 days.

L267 Where are these 95% CIs? Need to refer to Table 4 here.

L279 What is the time point for all these analyses? How many days after feeding on the bird?

L286 This way of presenting the data is very non-intuitive for the reader. Why not show the fraction of mosquitoes infected? The data are 0/1 and thus a logistic regression is more appropriate theoretically. See below for plots of the data and fitted models that are much easier to understand.

L336-7 This comparison is completely misdirected. The key difference between the competence estimates in the current study and that of ref 23 is not the assumption that a given viremia produces the same infectiousness. The key difference is that the viremias used in ref 23 come from Komar et al 2003 EID (which should be cited), in which viremias for grackles were 3-5 log10 higher than for robins, including a very high viremia on day 4 post-infection. The data in Fig 1 (and ref 14) suggests that in this study viremias were similar or slightly lower for grackles. So the main difference in competence values is only partly due to differences in the viremia-infectiousness relationship, and moreso due to differences in viremias observed in the experimental infections.

L351-3 This sentence is very strange in ignoring the last decade of work on West Nile virus. A half dozen studies have now shown that neither highly viremic hosts (crows, jays), nor common birds (house sparrows) play an important role in feeding mosquitoes. Instead, hosts that are highly preferred by feeding mosquitoes (primarily robins) are more important than all these species (often combined) and that these species are often uncommon (making up <10% of the birds at a site). See, for example:

Hamer, G. L., U. D. Kitron, T. L. Goldberg, J. D. Brawn, S. R. Loss, M. O. Ruiz, D. B. Hayes, and E. D. Walker. 2009. Host selection by Culex pipiens mosquitoes and West Nile virus amplification. American Journal of Tropical Medicine and Hygiene 80:268-278.

Kent, R., L. Juliusson, M. Weissmann, S. Evans, and N. Komar. 2009. Seasonal blood feeding behavior of Culex tarsalis (Diptera: Culicidae) in Weld County, Colorado, 2007. Journal of Medical Entomology 46:380-390.

Kilpatrick, A. M., P. Daszak, M. J. Jones, P. P. Marra, and L. D. Kramer. 2006. Host heterogeneity dominates West Nile virus transmission. Proceedings of the Royal Society B-Biological Sciences 273:2327-2333.

All these papers should be cited.

However, more broadly, in order to determine which hosts are important in infecting mosquitoes, it's critical to be able to quantify how infectious each species is. The work here suggests that more work needs to be done to determine if there is substantial differences among species in their infectiousness given the same viremia or if grackles are simply anomalous.

L355 This is incorrect. Robins are not particularly abundant. See comment and papers above. They are often relatively uncommon.

Statistical analyses

I downloaded the supplemental material and saved it as a .csv file. I changed the names of the columns to the following, in order (b/c R doesn't allow some of the symbols used):

Species

ID

Viremia

MosqStrain

PrevInf

Ninf

infected

notinfected

PrevDissInf

Ndissinf

Disseminated

I then fit models to the full dataset, as described above that included bird species, mosquito strain, and viremia, as well as the 3 way interaction. I also examined models with just a 2 way interaction between viremia and bird species. All code to replicate these analyses is given below.

First, I plotted the data and added fitted lines that reflect the 3-way interaction glm model below WITHOUT random effects which produced this figure:

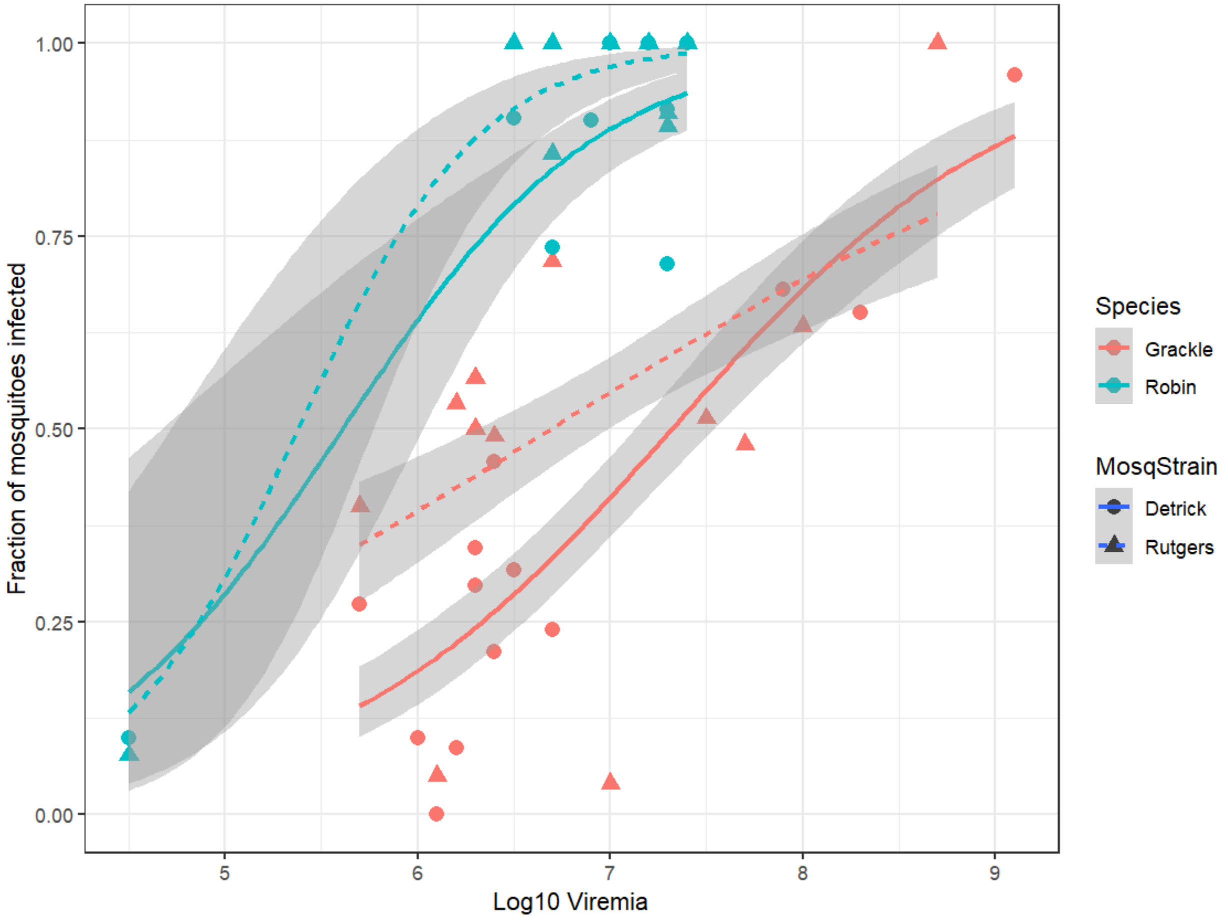

This figure initially supported the main conclusions of the study – that the fraction of mosquitoes infected differed among bird species and mosquito strains. This fitted model is here:

f1_3=glm(PrevInf~Viremia*Species*MosqStrain,family="binomial",weights=Ninf,data=m);summary(f1_3
)

	Estimate	Std. Error	z value	Pr(>|z|)
(Intercept)	-8.1779	0.8256	-9.905	< 2e-16
Viremia	1.1171	0.1164	9.6	< 2e-16
SpeciesRobin	-0.2138	2.3869	-0.09	0.928619
MosqStrainRutgers	3.9961	1.1528	3.466	0.000528
Viremia:SpeciesRobin	0.3789	0.3511	1.079	0.280554
Viremia:MosqStrainRutgers	-0.4921	0.1631	-3.016	0.002559
SpeciesRobin:MosqStrainRutgers	-7.1005	3.4968	-2.031	0.042299

Viremia:SpeciesRobin:MosqStrainRutgers	1.1331	0.5268	2.151	0.031471

Interestingly, it suggests that there is no intercept or slope difference for robins for Detrick mosquitoes, but there is a higher slope and lower intercept for robins for Rutgers mosquitoes.

However, this analysis fails to account for the repeated feedings on individual birds. When we fit a model that accounts for this (by including a random effect for individual bird) we get a much better fitting model, and we find that the conclusions are quite different:

f0_3=glmer(PrevInf~Viremia*Species*MosqStrain+(1|ID),family="binomial",weights=Ninf,data=m);summary(f0_3)

	Estimate	Std. Error	z value	Pr(>|z|)
(Intercept)	-10.693	1.63522	-6.539	6.19E-11
Viremia	1.49156	0.23442	6.363	1.98E-10
SpeciesRobin	0.05666	3.28649	0.017	0.9862
MosqStrainRutgers	2.67229	1.61211	1.658	0.0974
Viremia:SpeciesRobin	0.37029	0.47635	0.777	0.437
Viremia:MosqStrainRutgers	-0.27986	0.23637	-1.184	0.2364
SpeciesRobin:MosqStrainRutgers	-5.39683	3.79267	-1.423	0.1547
Viremia:SpeciesRobin:MosqStrainRutgers	0.87282	0.5723	1.525	0.1272

This model suggests that there isn't support for the 3 way interaction, and so we fit a model that just included the 2-way interaction between viremia and species and the main effect of mosquito species (this was supported by AIC over models with the 3 way interaction and other 2 way interactions). This best fitting model is here:

f0_2=glmer(PrevInf~Viremia*Species+MosqStrain+(1|ID),family="binomial",weights=Ninf,data=m);summary(f0_2)

	Estimate	Std. Error	z value	Pr(>|z|)
(Intercept)	-10.0493	1.5711	-6.396	1.59E-10
Viremia	1.3919	0.2233	6.233	4.58E-10
SpeciesRobin	-2.3269	2.7029	-0.861	0.3893
MosqStrainRutgers	0.8309	0.1608	5.166	2.39E-07
Viremia:SpeciesRobin	0.748	0.3913	1.912	0.0559

This model suggests that there is weak support that doesn't quite make the threshold for significance (P = 0.06) for robins having a higher slope. The contrast between this model and a similar model that doesn't have the random effect for individual bird suggests that most of the effect is due to differences among individual birds, not between species:

f1_2=glm(PrevInf~Viremia*Species+MosqStrain,family="binomial",weights=Ninf,data=m);summary(f1_2)

	Estimate	Std. Error	z value	Pr(>|z|)
(Intercept)	-6.60188	0.57846	-11.413	< 2e-16
Viremia	0.88874	0.08048	11.043	< 2e-16

SpeciesRobin	-3.4794	1.70962	-2.035	0.041832
MosqStrainRutgers	0.61268	0.13507	4.536	5.73E-06
Viremia:SpeciesRobin	0.88268	0.25495	3.462	0.000536

Specifically, the best fitting model (f0_2), bolded above, shows that the difference among species is 0.748 which is equivalent to a little more than half of one unit of log10 viremia (which has a slope of 1.3919). The standard deviation of the random effect (i.e. the variation among individual birds) is slightly larger than the effect of species (0.8027).

Thus, overall the data suggest that there may be a modest difference among species in the likelihood of infecting mosquitoes, but it is small relative to differences in viremia, and similar to differences among individuals.

(Note: All the analyses above were done with a binomial distribution and a logit link which is more appropriate than a probit link for this 0/1 data. However, I repeated the analysis using a probit link and the results are qualitatively identical).

<u>Analyses of Disseminated infections</u>

I also performed the same analyses on the data on disseminated infections. This dataset is much more limited since there was no data for about half of the feedings. However, it is much more epidemiologically relevant.

As above, I first plotted the data and added fitted lines that reflect the 3-way interaction glm model below WITHOUT random effects which produced this figure:

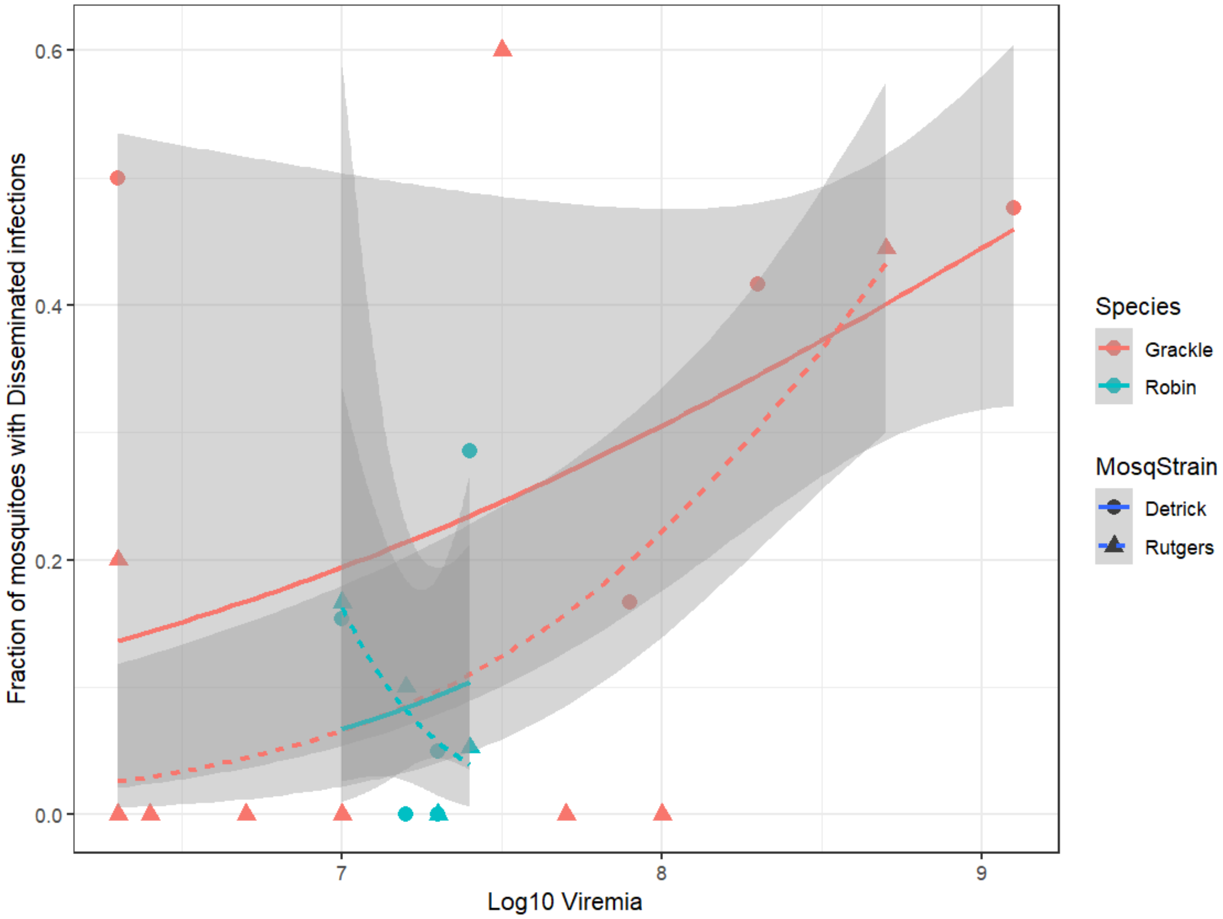

This data spans a much narrower viremia. The fitted model, even without random effects, suggests that the 3-way interaction wasn't needed:

f3=glm(PrevDissInf~Viremia*Species*MosqStrain,family="binomial",weights=Ndissinf,data=m);summary (f3)

	Estimate	Std. Error	z value	Pr(>|z|)
(Intercept)	-5.6313	3.5987	-1.565	0.118
Viremia	0.6012	0.414	1.452	0.146
SpeciesRobin	-5.375	24.7282	-0.217	0.828
MosqStrainRutgers	-6.8071	4.8309	-1.409	0.159
Viremia:SpeciesRobin	0.5953	3.3901	0.176	0.861
Viremia:MosqStrainRutgers	0.7972	0.5654	1.41	0.159
SpeciesRobin:MosqStrainRutgers	43.4095	37.6167	1.154	0.249
Viremia:SpeciesRobin:MosqStrainRutgers	-5.8843	5.1863	-1.135	0.257

I fit a model with random effects for individual bird, and just the 2 way interaction between bird species and viremia (the same as the best model above):

g0=glmer(PrevDissInf~Viremia*Species+MosqStrain+(1|ID),family="binomial",weights=Ndissinf,data=m);summary(g0)

	Estimate	Std. Error	z value	Pr(>|z|)
(Intercept)	-9.71323	2.28882	-4.244	2.20E-05
Viremia	1.07308	0.2624	4.089	4.32E-05
SpeciesRobin	13.23432	17.74155	0.746	0.456
MosqStrainRutgers	-0.05553	0.33207	-0.167	0.867
Viremia:SpeciesRobin	-1.88723	2.4399	-0.773	0.439

The model indicates that there is no evidence for a difference in disseminated infection between species. Neither the intercept or slope are remotely near significance. In contrast, there is strong evidence for an increase in disseminated infections with viremia.

<u>Full commented R code to reproduce the results above:</u>

```
lapply(c("ggplot2","lme4"),require,character.only=T) #load packages

setwd("c:/xxx/xxx/") #set wording directory

m=read.csv("VaughanData.csv")


#Infection data

#Plot of data w/ 3 way interaction model w/out random effects

ggplot(m, aes(y=PrevInf, x=Viremia,color=Species,shape=MosqStrain,
        linetype=MosqStrain,weight=Ninf)) +
  theme_bw()+geom_point(size=3)+labs(x = "Log10 Viremia", y = "Fraction of mosquitoes infected")+
  geom_smooth(method="glm",method.args=list(family="binomial"))


#3 way interaction models

#no random effects

f1_3=glm(PrevInf~Viremia*Species*MosqStrain,family="binomial",weights=Ninf,data=m);summary(f1_3)

#W random effects

f0_3=glmer(PrevInf~Viremia*Species*MosqStrain+(1|ID),family="binomial",weights=Ninf,data=m);summary(f0_3)

AIC(f1_3,f0_3) #mixed effects model is clearly superior
```

```
#2 way interaction models W random effects

f0_2=glmer(PrevInf~Viremia*Species+MosqStrain+(1|ID),family="binomial",weights=Ninf,data=m);summary(f0_2)

AIC(f0_2,f0_3) #2-way is better than 3-way

#other 2-way interaction models

f0_2_b=glmer(PrevInf~Viremia*Species+Viremia*MosqStrain+Species*MosqStrain+(1|ID),family=binomial,weights=Ninf,data=m);summary(f0_2_b)

f0_2_c=glmer(PrevInf~Viremia*Species+Viremia*MosqStrain+(1|ID),family=binomial,weights=Ninf,data=m);summary(f0_2_c)

AIC(f0_2,f0_2_b,f0_2_c) #f0_2 is best


#Disseminated infections

#Plot of data w/ 3 way interaction model w/out random effects

ggplot(m[complete.cases(m),], aes(y=PrevDissInf, x=Viremia,color=Species,shape=MosqStrain,

                              linetype=MosqStrain,weight=Ndissinf)) +

  theme_bw()+geom_point(size=3)+labs(x = "Log10 Viremia", y = "Fraction of mosquitoes with Disseminated infections")+

  geom_smooth(method="glm",method.args=list(family="binomial"))


#3 way interaction models

#no random effects

g3=glm(PrevDissInf~Viremia*Species*MosqStrain,family="binomial",weights=Ndissinf,data=m);summary(g3)

#2 way interaction model W random effects

g0=glmer(PrevDissInf~Viremia*Species+MosqStrain+(1|ID),family="binomial",weights=Ndissinf,data=m);summary(g0)
```
