# Supplementary material for: Bird species define the relationship between West Nile viremia and infectiousness to Culex pipiens mosquitoes
Source: PLoS Negl Trop Dis. 2022 Oct 6;16(10):e0010835. doi: 10.1371/journal.pntd.0010835 (PMC9578590; doi:10.1371/journal.pntd.0010835)
Supplement: Attachment — Submitted filename: 2022-PLoS NTD-Grackle VS Robin Infectivity-For submission.rev.docx [file pntd.0010835.s002.docx]

**Bird species define the relationship between West Nile viremia and infectiousness to *Culex pipiens* mosquitoes**

Jefferson A. Vaughan^1^, Michael J. Turell^2,3^

^1^ Department of Biology, University of North Dakota, Grand Forks, North Dakota, United States of America

^2^ Virology Division, US Army Medical Research Institute of Infectious Diseases, Fort Detrick, Maryland, United States of America

^3^ Current Address: VectorID LLC, Frederick, Maryland, United States of America

Keywords: American Robin, arbovirus, Common Grackle, *Culex pipiens*, host competence index, viremia, West Nile virus

Short Title: Viremias in different bird species infect mosquitoes differently

Corresponding author: Email: [jefferson.vaughan@und.edu](mailto:jefferson.vaughan@und.edu)

**ABSTRACT**

The transmission cycle of West Nile virus involves multiple species of passerine birds. The relative importance to overall transmission of various bird species often is inferred from the level and duration of viremia that a bird species produces upon infection with West Nile virus (WNV). Present inferences for the infectivity of WNV-infected birds to mosquitoes are based on a laboratory model system whereby groups of mosquitoes were fed on viremic baby chickens. However, recent reports utilizing artificial membrane feeding techniques suggest that the source and condition of blood in which arboviruses are presented to mosquitoes can significantly alter the infectiousness of arbovirus to mosquitoes. To test this with live hosts, we conducted a series of mosquito feedings with *Culex* *pipiens* on WNV-infected American Robins and Common Grackles with a wide range of viremias. Mosquitoes were assayed for infection 3 to 7 days after feeding. Viremic Robins were significantly more infectious (LC-50 = log_10_ 5.6 plaque-forming units/ml) than were viremic Grackles (LC-50 = log_10_ 7.4 plaque-forming units /ml). At equivalent levels of viremia, Robins always infected more mosquitoes than did Grackles. We conclude that the infectiousness of viremic wild birds to mosquitoes cannot be extrapolated based merely on peak viremia.

**AUTHOR SUMMARY**

We infected two bird species, American Robins and Common Grackles, with West Nile virus and then fed *Culex pipiens* mosquitoes on these birds. We found that, despite having equivalent or lower levels of viremia, Robins infected more mosquitoes than did Grackles. The reasons for this are not presently known, but our results indicate that the relationship between host viremia and infectiousness is not always straight forward. To gain precise information for a mosquito-borne arbovirus system, it is best to conduct mosquito feedings on live viremic hosts rather than extrapolate the infectiousness of a viremic host solely from its viremia.

**Introduction**

In a typical mosquito-borne arboviral transmission cycle, virus moves from a viremic vertebrate host into a susceptible mosquito vector and, after a period of time, back again into a new, immunologically naïve vertebrate host that subsequently becomes viremic. The interaction between virus and mosquito is crucial to the perpetuation of the cycle. For transmission to proceed, the virus must be capable of infecting and propagating within the mosquito midgut, disseminating throughout the mosquito hemocoel, and ultimately infecting the salivary glands to be secreted in mosquito saliva, the next time the mosquito probes and feeds. Fortunately, this series of events does not happen with every combination of mosquito and virus. Instead, the efficiency of the infection process within a mosquito is specific to the particular mosquito species and arbovirus involved and can range from species combinations wherein the infection aborts at some point (*i.e.,* non-vector species) to species combinations wherein the process culminates in virus transmission (*i.e*., vector). Defining the efficiencies of infection for various arbovirus-mosquito species combinations is fundamental to the concept of vector competence and has been the subject of study for many years. For each specific arbovirus-mosquito species combination, there exist modulating factors that either increase or decrease infection efficiency. Modulating factors can be categorized as intrinsic (*e.g.,* mosquito genetics, virus strain), abiotic (*e.g*., environmental temperature), or biotic (*e.g.,* adventitious viruses, *Wolbachia*, microsporidia, *etc*.).

Perhaps the most important modulating factor defining the efficiency of arboviral infection within mosquitoes is the quantity of virus ingested. This is defined by the level of viremia within a vertebrate host at the time of mosquito feeding. As is the case with different mosquito species, the efficiency of arbovirus infection varies among vertebrate host species, ranging from vertebrate-virus species combinations wherein the virus fails to replicate (*i.e.,* “dead-end” host) to vertebrate-virus species combinations wherein virus replicates efficiently and achieves high quantities in the blood (*i.e.,* “amplifying host”). Therefore for arbovirus transmission cycles involving multiple vertebrate host species, not all vertebrate species may contribute equally to the perpetuation of the cycle. Not surprisingly, the relative contribution of a host species (i*.e*., reservoir competence) traditionally has been inferred from its characteristic viremia – that is, vertebrate species developing high viremias are more important in the transmission cycle than vertebrate species developing low viremia [1]. But a closer examination of the literature suggested that it may be more nuanced than merely defining vertebrate viremia.

Several studies have shown that the source and condition of blood in which arboviruses are presented to mosquitoes can significantly affect the efficiency of mosquito infection. Most of these studies have utilized *in vitro* mosquito feeding systems – *e.g*., blood-soaked gauze, membrane feeders, *etc*. The advantages of these systems are that the virus concentration and diluent composition offered to mosquitoes can be precisely controlled. Using blood-soaked gauze, Hardy and Reeves [2] first reported that Saint Louis encephalitis virus was more infectious to *Cx. tarsalis* mosquitoes when presented in defibrinated blood obtained directly from a viremic chicken than was the same amount of virus obtained from suckling mouse brains and added to normal, defibrinated rabbit or chicken blood. Similarly, Huang *et al*. [3] reported that Zika virus mixed in defibrinated whole sheep blood was significantly more infectious to *Aedes aegypti* mosquitoes (64% infected, n=82) than the same concentration of Zika virus mixed in bovine serum albumin (27% infected, n=82). They suggested that cellular components of the blood meal may be important in the virus infection process. More recently, Abbo *et al*. [4] reported that Usutu virus mixed in whole human blood (anticoagulation method not stated) was significantly more infectious to two biotypes of *Cx. pipiens* (66% infected, n=234) than the same concentration of Usutu virus mixed in whole chicken blood (49%, n=283). Their results suggest that in addition to cellular components, there may be soluble factors involved with the virus infection process. The most detailed study describing this phenomenon was that of Marchi *et al*. [5]. They examined the infectivity of African horse sickness virus (Reoviridae) to the midge vector, *Culicoides variipennis*. Infection rates in midges fed 5 days earlier on virus at 5 x 10^7^ tissue culture infectious dose 50% per ml differed markedly depending on the blood source to which the virus had been added. When added to heparinized horse or dog blood, the virus infected 51% (n=933) and 41% (n=209) of the midges respectively, but when added to heparinized sheep or cow blood, the same concentration of virus infected only 26% (n=160) and 11% (n=160) of the midges respectively. When purified virus was incubated overnight in horse or dog sera, gel electrophoresis demonstrated that the outer capsid protein VP2 of the virus was cleaved, whereas the VP2 protein remained intact after overnight incubation with cow or sheep sera. Furthermore, pretreatment of virus with chymotrypsin not only cleaved the viral capsid VP2 but when mixed with cow serum and fed to midges, increased the infectivity of cow/virus bloodmeals to midges from 11% (n=160) to 51% (n=60). This suggested that in the case of African horse sickness virus and midge vectors, species-specific serum proteases play an important role in determining virus infectivity to the vector.

*In vitro* feeding systems have clear advantages but also have the disadvantage that they require defibrination or addition of anti-clotting agents (*e.g*., heparin) to the blood. Without normal blood clotting, ingested virions remain distributed randomly throughout the blood bolus instead of being pushed outwards towards the periphery as the result of normal fibrin formation [6]. Concentration of virions on the peripheral portion of a blood meal during clotting allows more virions to contact receptor sites on the midgut lumen. Not surprisingly, use of live viremic hosts typically results in significantly increased mosquito infection rates compared to *in vitro* feeding systems [7-9]. Live host feedings more closely represent the natural situation but can be more challenging because viremias in a live host cannot be controlled. Comparing arboviral infectivity of different host species using live host feedings often require mosquito feedings on multiple animals and/or multiple times throughout the course of viremia, in the hope of obtaining matching viremias in two host species at the time of mosquito feeding. Few studies have done this. As part of a larger study, Reisen *et al*. [10] used restrained, viremic birds to infect mosquitoes with West Nile virus (WNV). When a population of *Cx. tarsalis* from Kern Co., CA, fed on a house finch (*Carpodacus mexicanus*) with a titer of WNV of 5.4 log_10_ plaque-forming units (PFU)/ml, and similarly on a 7-day old chicken (*Gallus domesticus*) with a slightly higher titer of WNV of 5.7 log_10_ PFU/ml, the resulting mosquito infection rate in mosquitoes fed on the viremic finch (94%, n=17) was significantly higher than in mosquitoes fed on the viremic chick (13%, n=16) (p<0.00001). Although this represents only a single comparative feeding, their experiment (together with *in vitro* feeding studies cited above) suggest that viremic blood in different bird species can significantly alter the infectivity of WNV to *Culex* mosquitoes feeding on viremic birds.

Our study expands on the observation by Reisen *et al.* [10] by conducting multiple mosquito feedings on two species of passerine birds over a range of WNV viremia to determine if the relationship between host viremia and mosquito infection differs for different host species.

**Materials and Methods**

**Birds**

American Robins (*Turdus migratorius*) were captured from Roseau and Pennington Co., MN, using mist nets. Common Grackles (*Quiscalus quiscala*) were captured from Grand Forks Co., ND, using baited ground traps. Birds were maintained in an outdoor aviary at the University of North Dakota where they were screened for blood parasites and antibodies to WNV [11-13]. Six Robins and nine Grackles were selected for use and transported during three separate trips in modified pet carriers via commercial airline from Grand Forks, ND, to Washington DC and via automobile to the United States Army Medical Research Institute of Infectious Diseases (USAMRIID) in Frederick, MD. Birds were housed in standard bird cages for an acclimatization period of ≥ 24 h prior to infection with WNV. Because blood parasitemia is a natural condition for these bird species, parasitemias were recorded but birds were not treated with anti-helminthic or anti-protozoal drugs prior to use. The inclusion criterion was simply testing negative to WNV antibodies. However, after experiments were complete, data from one of the nine original Grackles had to be excluded from this report because it was discovered that a pre-existing microfilarial infection in this bird significantly increased WNV infectivity and dissemination in mosquitoes that fed on this bird [14]. Microfilariae present in four of the other Grackles did not affect the course of WNV infectivity in mosquitoes, compared to mosquitoes fed on non-microfilaremic Grackles. Similarly, blood parasites (*i.e.,* *Plasmodium*, trypanosomes, microfilariae) in the Robins did not affect WNV infectivity to mosquitoes [14].

**Mosquitoes**

Two strains of *Cx. pipiens* were used. The ‘Rutgers’ strain originated from larvae collected in the 1980’s at East Brunswick, NJ (F>200). The ‘Area B’ strain originated from larvae collected in 2010 at Fort Detrick, MD (F~30).

**Virus**

The 397-99 strain of WNV was used throughout. This strain originated from an infected crow in 1999 at the Bronx Zoo [15].

**Mosquito Infections**

Birds were inoculated subcutaneously with 0.2 ml of WNV (6.1 log_10_ PFU/bird) between 1700 and 1930 h. At intervals of 30, 54 and 78 h after WNV infection, birds were anesthetized and placed through the cotton stockinette of cylindrical screen-topped cardboard cages containing *ca*. 50 mosquitoes. Mosquitoes were allowed to feed directly on the birds for *ca*. 30 minutes. Unfed mosquitoes were removed. Three fully engorged mosquitoes from each cage were removed, triturated, and tested on Vero cells by plaque assay [15] to determine the viremia at the time of feeding. Viral titers were expressed as log_10_ PFU/ml. Subsamples of mosquitoes were removed 3, 4, 5, and 7 days later, triturated individually in 1 ml of diluent, and stored at –80^o^C until tested for virus by plaque assay.

**Data Analyses**

Rates of viral infection were compared by chi square analyses with Yates correction (Statistix, Tallahassee, FL) among groups of mosquitoes that fed on birds with equivalent viremia. To examine the relationship of bird viremia and mosquito infection, dose-response equations for both bird species were calculated using probit regression analyses with log_10_ viremia as the independent variable and percentage mosquito infection transformed to probits as dependent variables (IBM SPSS Statistics, Armonk NY). Statistical differences between regression lines were assessed by comparing regression coefficients (*i.e*., slopes, *b*) with t-tests [16]. Comparison of the dose-response curves of mosquitoes fed on Robins versus Grackles was complicated by the observation that when the two strains of mosquitoes fed on Grackles at equivalent viremias, there was a difference in strain susceptibility at moderate host viremia. To determine if these “point differences” between the two mosquito strains fed on viremic Grackles were of sufficient magnitude to statistically alter the overall relationship between Grackle viremia and mosquito infection, a probit regression was computed for the Area B strain and, separately for the Rutgers strain of *Cx. pipiens* fed on Grackles. There was no significant difference between slopes for Area B (*b*=0.25±0.10) and Rutgers (*b*=0.58±0.08) (t=0.29, df=14, p=0.77) and thus infection data for the two strains feeding on Grackles were pooled. Combining both mosquito strains, probit regression statistics were then calculated and slopes compared between the viremia-infectiousness relationships of Robins (=8 viremias, ranging from log_10_ 4.5 to 7.4) versus Grackles (14 viremias, ranging log_10_ 5.7 to 8.3). A third probit regression was calculated for *Cx. pipiens* fed on 2 to 5-day-old WNV-infected chickens (*Gallus domesticus*) (11 viremias, ranging from log_10_ 4.0 to 8.2 PFU/ml) [15,17,18] and compared to those from Robins and Grackles. The 0.05 level of significance was used throughout.

To compare host competence between bird species, a competence index, *C_i_*, was calculated for both species as the product of ‘*infectiousness’* (*i*) and ‘*duration of infectivity*’ (*d*): *C_i_=(i)(d)*, where *i* represents the proportion of mosquitoes that became infected after feeding during the period of infectious viremia and *d* represents the average number of days of infectious viremia [19].

**Ethics Statement**

Research was conducted under IACUC approved protocols from the University of North Dakota and the USAMRIID in compliance with the Animal Welfare Act and other Federal regulations relating to the experimental use of vertebrate animals. Collection, transport, and experimentation with migratory birds were conducted under the approval of U.S. Fish & Wildlife Service scientific collection permit MB072162 and collecting permits from the state wildlife agencies of North Dakota and Minnesota.

**Results**

**Viremia Profiles**

The course and magnitude of WNV viremia for Robins and Grackles were similar [14]. Peak viremia in six Robins occurred 1 to 2 days after inoculation and ranged from 6.5 to 7.4 log_10_ PFU/ml (average 7.1 ± 0.3). Similarly, peak viremias in eight Grackles occurred on days 1 to 2 after inoculation but their magnitudes were more variable, ranging from 6.3 to 8.3 log_10_ PFU/ml (average 6.9 ± 0.8). Viremia in both bird species began to subside by Day 3.

**Effect of Mosquito Strain**

A total of 1,173 *Cx. pipiens* mosquitoes were tested by plaque assay for WNV infection. Of these, 399 fed on Robins (187 Area B strain, 212 Rutgers strain), and 774 fed on Grackles (416 Area B strain, 358 Rutgers strain). To determine if the two mosquito strains differed in susceptibility, mosquito infection rates were compared where the two strains fed concurrently on the same bird or a bird of the same species with the same viremia. In instances where mosquito strains fed on two con-specific birds having the same viremia, mosquito infection data for respective mosquito strains were combined. The Area B strain was more susceptible to infection than the Rutgers strain, but the magnitude of difference was influenced by bird species (Table 1). For Robins, there were seven levels of viremia in which both mosquito strains fed concurrently. Only one (6.7 log_10_ PFU/ml) resulted in a significant difference between mosquito strains. For Grackles, there were four levels of viremia in which both mosquito strains fed concurrently. The Area B strain was significantly more susceptible than Rutgers strain in two of the four concurrent feedings (6.2 and 6.3 log_10_ PFU/ml) and nearly statistically significant (p=0.07) in a third (6.4 log_10_ PFU/ml), and the fourth, while not significant, the Area B mosquitoes had an infection rate 50% higher than the Rutgers strain, but the sample size was small (Table 1). The difference in virus concentration between 6.2 and 6.4 log_10_ PFU/ml is small (< 2-fold). When infection data for these viremia levels were combined, the infection rate for the Area B strain (51%, n=196) was highly significantly greater than for the Rutgers strain (22%, n=74) (p<0.0001).

**Table 1.** West Nile virus infection rates in two strains of *Culex pipiens* mosquitoes 5 to 7 days after feeding concurrently on the same viremic bird. Mosquito infection data are pooled in instances where two birds had the same viremia.

|  | |  | |  | | Mosquito Infection Rate  (number tested) | |  |
| --- | --- | --- | --- | --- | --- | --- | --- | --- |
| Bird Species | Bird(s) ID # | | Host Viremia  (log_10_ PFU/ml) | | Area B strain | | Rutgers strain | p-value |
| American Robin | 67 | | 4.5 | | 8% (13) | | 10% (10) | 0.692 |
|  | 67 | | 6.5 | | 100% (13) | | 90% (31) | 0.612 |
|  | 3, 89 | | 6.7 | | 100% (53) | | 77% (48) | 0.002 |
|  | 108 | | 7.0 | | 100% (11) | | 100% (23) | 1.000 |
|  | 52 | | 7.2 | | 100% (20) | | 100% (20) | 1.000 |
|  | 89, 108 | | 7.3 | | 90% (39) | | 84% (56) | 0.612 |
|  | 55 | | 7.4 | | 100% (38) | | 100% (14) | 1.000 |
| Common Grackle | 152 | | 5.7 | | 40% (20) | | 27% (11) | 0.479 |
|  | con | | 6.2 | | 53% (15) | | 9% (23) | 0.007 |
|  | con, 53 | | 6.3 | | 54% (93) | | 32% (32) | 0.046 |
|  | 53, 29 | | 6.4 | | 47% (88) | | 21% (19) | 0.074 |

**Comparative Infectivity Between Bird Species**

To examine comparative infection in mosquitoes fed on the two bird species, the infection rates of mosquitoes fed on Robins were paired with infection rates of mosquitoes of the same mosquito strain fed on Grackles, where the viremias were similar at the time of mosquito feeding (Table 2). There were two exact matches (using Area B strain) where individual birds of both species had matching viremias of 6.5 and 6.7 log_10_ PFU/ml (see pairings 1 & 2, Table 2). In both pairings, viremic Robins infected a significantly greater proportions of mosquitoes than did Grackles with matching viremia (p<0.0001). Three other pairings were examined wherein mosquitoes of the same strain fed on viremic Grackles that had four to 10-times more virus than Robins (see pairings 3, 4 & 5, Table 2). In each of these pairings, the viremic Robins infected significantly greater proportions of mosquitoes than did the viremic Grackles (p<0.05), despite the mosquitoes ingesting less WNV from the Robins that from the Grackles. When host competence indices were compared between bird species using the method described by Komar *et al.* [19], Robins (*C_robin_* = 1.38) were two-fold more competent as hosts for WNV than were Grackles (*C_grackle_* = 0.66) (Table 3). This reflected the large difference in their average daily infectiousness (*i*), but not the average durations of their infectious period (*d*).

**Table 2**. West Nile-infected American Robins are more infectious to *Culex pipiens* mosquitoes (both strains) than are Common Grackles with equivalent or higher viremias.

| Pairing | Mosquito Strain | Bird Species | No. Birds | Viremias (log_10_ PFU / ml) | Mosquito Infection Rate (number tested) | p-value |
| --- | --- | --- | --- | --- | --- | --- |
| 1 | Area B | Robin | 1 | 6.5 | 100% (13) | <0.0001 |
|  |  | Grackle | 1 | 6.5 | 32% (60) |  |
| 2 | Area B | Robin | 2 | 6.7 | 100% (53) | <0.0001 |
|  |  | Grackle | 2 | 6.7 | 49% (103) |  |
| 3 | Area B | Robin | 3 | 7.3 – 7.4 | 95% (77) | <0.0001 |
|  |  | Grackle | 1 | 8.0 | 63% (60) |  |
| 4 | Rutgers | Robin | 2 | 6.7 | 77% (48) | 0.0058 |
|  |  | Grackle | 1 | 7.7 | 48% (50) |  |
| 5 | Rutgers | Robin | 2 | 7.3 | 84% (56) | 0.0332 |
|  |  | Grackle | 1 | 8.3 | 65% (63) |  |

**Table 3**. Comparative host competence indices for American Robins and Common Grackles to serve as amplifying hosts for West Nile virus.

| Bird Species | Mosquito Species | No. Birds | Days of Infectiousness  (*d*) | Proportion Mosquitoes Infected  (*i*) | Host competence (*C_i_*) |
| --- | --- | --- | --- | --- | --- |
| American Robin | *Culex pipiens* | 6 | 1.58 | 0.87 | 1.38 |
| Common Grackle | *Culex pipiens* | 8 | 1.77 | 0.37 | 0.66 |

**Viremia-Infectivity Relationship**

The estimated concentrations of viremia needed to infect 50% of the mosquitoes (*i.e*., the IC-50’s) were similar between baby chickens (5.7 log_10_ PFU/ml) and adult Robins (5.6 log_10_ PFU/ml), with overlapping confidence intervals. The IC-50 for viremia in adult Grackles (7.4 log_10_ PFU/ml) was 25 to 60-fold higher and 95% confidence intervals did not overlap, indicating a statistically significant difference. As viremia increased, the virions produced in Grackles became less infectious to mosquitoes than virions produced in either Robins or chickens (Fig. 1). This was reflected in the significantly higher amount of virus (*ca*. 5,000-fold more) needed to infect 90% of mosquitoes fed on viremic Grackles versus baby chickens and Robins (Table 4). The slope for the viremia-infectiousness relationship of mosquitoes fed on viremic Grackles (*b*=0.39 ± 0.06) was significantly less than for mosquitoes fed on either viremic Robins (*b*=1.04 ± 0.13) (t=2.4, df=18, p=0.025) or viremic baby chickens (*b*=1.16 ± 0.07) (t=2.6, df=21, p=0.016) (Table 4). There was no significant difference in the slopes for the viremia-infectiousness relationship between mosquitoes fed on either Robins or baby chickens (t=-0.67, df=15, p=0.513).

**Table 4**. Amount of West Nile virus (log_10_ plaque-forming units) per milliliter of blood in three bird species required to infect *Culex pipiens* mosquitoes at moderate (50%), and high (90%) rates of infection.

| Bird infectivity | Probability of mosquito infection after feeding on viremic bird | Predicted virus concentration  (95% confidence interval)  expressed as log_10_ PFU/ml | | |
| --- | --- | --- | --- | --- |
|  |  | Baby chicken* | American Robin | Common Grackle |
| Moderately Infectious | 50%  (ID-50) | 5.7  (5.3, 6.1) | 5.6  (1.3, 6.3) | 7.4  (6.8, 8.9) |
| Highly Infectious | 90%  (ID-90) | 6.8  (6.4, 7.5) | 6.8  (6.1, 7.8) | 10.6  (9.0, 19.5) |
| Regression slope (95% CI) | | 1.2 (1.0, 1.3) | 1.0 (0.8, 1.3) | 0.4 (0.3, 0.5) |
| Number of viremias (range) used in probit regression | | 11  (4.0 – 8.2) | 8  (4.5 – 7.4) | 14  (5.7 – 8.3) |
| Total mosquitoes tested | | 675 | 399 | 774 |

*data from [15,17,18].

**Figure 1. Dose-infection response of *Culex pipiens* mosquitoes to ingested West Nile virus when fed on viremic birds; American Robins (	), Common Grackle (	), and 3-to-6-day-old chickens ( X ) [15,17,18]**.

**Discussion**

American Robins and Common Grackles experienced similar levels of viremia when experimentally infected with WNV. However, Robins were found to be significantly more infectious to vector mosquitoes than Grackles – even when Robins had substantially lower viremia than Grackles (Table 2). The mechanism(s) responsible for different infectiousness of these two bird species is unknown, but it appears unlikely that the increased infectiousness of Robins resulted from co-infection of blood parasites [14]. More likely, different infectiousness of the two bird species resulted from innate differences in their blood and how the blood behaved once inside the mosquito midgut. There are at least two possibilities, neither mutually exclusive. First, the WNV virions may be rendered less infective in Grackle sera due to the activity (or lack thereof) of species-specific serum proteases, similar to that described for African horse sickness virus in dog versus horse sera [5]. Alternatively, the kinetics and/or intensity of fibrin formation in the sera of viremic Grackles may be different such that ingested WNV virions are not pushed to the periphery of blood meals within engorged *Cx. pipiens* mosquitoes to the same degree as in Robin sera. Reduced localization of virus to the periphery of blood meals would lead to a reduced number of contacts between ingested virions and midgut epithelium, resulting in lowered mosquito infection rates [6]. The notion that the viremic state can alter blood meal clotting is supported by the observation that dengue virus (also a flavivirus) alters the kinetics and intensity of fibrin formation within human sera of dengue patients [20].

Regardless of the mechanism involved, our results complicate the concept of vertebrate host competence, defined as the relative infectiousness of a vertebrate host species to mosquitoes. Originally developed for eastern equine encephalitis virus in European Starlings (*Sturnus vulgaris*) with *Culiseta melanura* mosquitoes, Komar *et al*. [19] proposed a simple calculation to derive a numerical value for host competence – the reservoir competence index (*C_i_*) – which is the product of the average daily host infectiousness (*i*) to mosquitoes and its duration in days (*d*). This index has become a critical component in estimating relative contributions of different host species in arbovirus transmission cycles in nature [21] and in scenario planning for predicting future invasive arbovirus transmission yet to occur [22].

For the index to be useful, the average daily infectiousness component (*i*) of the index must be determined experimentally. This is done by feeding mosquitoes on a range of virus concentrations and subsequently determining the proportion of mosquitoes infected at each concentration. The results are then used to construct a dose-response equation, making it possible to estimate the proportion of infected mosquitoes generated at different host viremias. Importantly, the viremia/infectiousness relationship must be determined experimentally by feeding mosquitoes on live viremic hosts, not *in vitro* methods (*e.g*., membrane feeders) because the use of defibrination or anticoagulants in artificial blood meals invariably reduce mosquito infectivity [7-9] and will lead to systemic underestimation of host competency indices.

Host competency indices for WNV in *Cx. pipiens* were calculated and compared among 53 vertebrate species by Kilpatrick *et al*. [23]. In this case, the infectiousness component (*i*) of the index was based on the viremia-infectiousness relationship of *Cx. pipiens* feeding on viremic 1-to-5-day old chickens from previously-published data [15,18,24]. No data from mosquito feedings on other bird species were available. Using the viremic profiles of the host species and the viremia-infectiousness equation based on baby chickens, Kilpatrick *et al*. [23] estimated the host competence index for Grackles (*C*_grackle_ ~ 1.7) to be substantially higher than for Robins (*C*_robin_ ~ 1.1). Based on actual mosquito feedings on these two bird species, we found that the competence index for Grackles (*C_grackle_* = 0.66) was half that of Robins (*C_robin_* = 1.38) (Table 3). Clearly, when baby chickens were used to define viremia-infectiousness relationship, Grackles were over-rated as competent hosts for WNV. Probit regression analyses indicated that the WNV viremia/infectiousness relationship of the baby chicken/*Cx. pipiens* model system was a reasonable surrogate for Robins but not for Grackles (Fig. 1, Table 4). Infectiousness of Grackles to *Cx. pipiens* was innately lower than Robins. Even at the highest viremias observed (8.0 to 8.3 log_10_ PFU/ml), Grackles never infected more than 65% of *Cx. pipiens*, whereas Robins with much lower viremias (6.5 to 7.0 log_10_ PFU/ml), infected 77 to 100% of *Cx. pipiens*.

Undoubtedly, mosquito feedings on viremic live birds will establish more accurate indices of host competency for WNV. But these types of experimental infections present challenges in terms of logistics, securing federal and state collecting permits, determining appropriate anesthesia or restraining methods, addressing compliance/biosafety issues, *etc*. From a practical standpoint, this level of stringency is more realistically done for high-priority bird species – *i.e*., birds known to produce high viremia (*e.g*., crows, jays) or are numerous, associated with humans, and commonly fed on by vector mosquitoes (*e.g*., house sparrows). Experimental mosquito feeding on viremic live birds may be less warranted for species that produce low viremia ≤ 4.6 log_10_ PFU/ml (*e.g*., pigeons, adult chickens) or are uncommon.

In terms of vertebrate amplification of WNV, Robins are recognized as one of the most important amplifying species in eastern North America due to their abundance and observations that *Cx. pipiens* mosquitoes tend to feed preferentially on Robins – especially during the early part of the transmission season [21,25-28]. The present study demonstrates that viremic Robins are also more innately infectious to *Cx. pipiens* than are viremic Grackles, further underscoring the importance of Robins as key amplifying hosts for WNV. More generally, our studies demonstrate that for WNV (and possibly other arboviruses), the relationship between host viremia and the efficiency by which a viremic host infects mosquitoes is not fixed and cannot be determined solely by measuring host viremia. The relationship will also depend on the host species involved.

**Acknowledgments**

Students in the Vaughan laboratory helped capture and screen birds. Ms. Danielle Kvasager and Mr. Chad Stromlund captured American Robins and screened them for microfilariae and trypanosomes. Ms. Sarina Bauer assisted with the molecular diagnosis of hemosporidian parasites. Mr. Lei Guo assisted with immunoassays to screen birds for antibodies to WNV. Technicians and research fellows in the Turell laboratory assisted with experimental procedures. Dr. Elizabeth Andrews and Ms. Juantia Hinson conducted mosquito trituration and plaque assays. Ms. Denise Nash helped rear the mosquitoes used in this study.

Disclaimers:

Opinions, interpretations, conclusions, and recommendations expressed in this article are those of the authors and do not reflect the official policy of the Department of Army, Department of Defense, or the U.S. Government.

**References**

1. Lord CC, Rutledge R, Tabachnick WJ. Relationship between host viremia and vector susceptibility for arboviruses. J Med Entomol. 2006;43: 623-630 doi: 10.1603/0022-2585(2006)43[623:rbhvav]2.0.co;2.
2. Hardy JL, Reeves WC. Experimental studies on infection in vectors. In: Reeves WC, editor. Epidemiology and control of mosquito-borne arboviruses in California, 1943-1987. Sacramento: California Mosquito and Vector Control Association; 1990. pp. 145-253.
3. Huang Y-JS , Lyons SAC, Hsu W-W, Park SL, Higgs S, Vanlandingham DL. Differential outcomes of Zika virus infection in *Aedes aegypti* orally challenged with infectious blood meals and infectious protein meals. PLoS One. 2017;12: e0182386. doi: 10.1371/journal.pone.0182386.\
4. Abbo SR, Visser TM, Koenraadt CJM, Pijlman GP, Wang H. Effect of blood source on vector competence of *Culex pipiens* biotypes for Usutu virus. Parasit Vectors. 2021;14: 194. doi: 10.1186/s13071-021-04686-6.
5. Marchi PR, Rawlings P, Burroughs JN, Wellby M, Mertens PP, Mellor PS, *et al*. Proteolytic cleavage of VP2, an outer capsid protein of African horse sickness virus, by species-specific serum proteases enhances infectivity in *Culicoides.* J Gen Virol. 1995;76: 2607-2611. doi: 10.1099/0022-1317-76-10-2607.
6. Weaver SC, Scott TW, Lorenz LH, Repik PM. Detection of eastern equine encephalomyelitis virus deposition in *Culiseta melanura* following ingestion of radiolabeled virus in blood meals. Am J Trop Med Hyg. 1991;44: 250-259. doi: 10.4269/ajtmh.1991.44.250
7. Turell MJ. Reduced Rift Valley fever virus infection rates in mosquitoes associated with pledget feedings. Am J Trop Med Hyg. 1989;39: 597-602. doi: 10.4269/ajtmh.1988.39.597.
8. Meyer RP, Hardy JL, Presser SB. Comparative vector competence of *Culex tarsalis* and *Culex quinquefasciatus* from the Coachella, Imperial, and San Joaquin Valleys of California for St. Louis encephalitis virus. Am J Trop Med Hyg. 1989;32: 305-11. doi: 10.4269/ajtmh.
9. Roundy CM, Azar SR, Rossi SL, Huang JH, Leal G, Yun R, *et al*. Variation in *Aedes aegypti* mosquito competence for Zika virus transmission. Emerg Infect Dis. 2017;23: 625-632. doi: 10.3201/eid2304.161484.
10. Reisen WK, Fang Y, Martinez VM. Avian host and mosquito (Diptera: Culicidae) vector competence determine the efficiency of West Nile and St. Louis encephalitis virus transmission. J Med Entomol. 2004;42: 367-375. doi: 10.1093/jmedent/42.3.367.
11. Collins JD. The detection of microfilariae using the capillary hematocrit tube methods. Trop Animal Health Prod. 1971;3: 23-25.
12. Blitvich BJ, Bowen RA, Marlenee NL, Hall RA, Bunning ML, Beaty BJ. Epitope-blocking enzyme-linked immunosorbent assays for detection of West Nile virus antibodies in domestic mammals. J Clin Microbiol. 2003;41: 2676-2679.
13. Hellgren O, Waldenstrom J, Bensch S. A new PCR assay for simultaneous studies of *Leucocytozoon*, *Plasmodium*, and *Haemoproteus* from avian blood. J Parasitol. 2004;90: 797-802.
14. Vaughan JA, Hinson J, Andrews ES, Turell MJ. Pre-existing microfilarial infections of American Robins (Passeriformes: Turdidae) and Common Grackles (Passeriformes: Icteridae) have limited impact on enhancing dissemination of West Nile Virus in *Culex pipiens* mosquitoes (Diptera: Culicidae). J Med Entomol. 2021;58: 1389-1397. doi: 10.1093/jme/tjaa261.
15. Turell MJ, O'Guinn M, Oliver J. Potential for New York mosquitoes to transmit West Nile virus. Am J Trop Med Hyg. 2000;62: 413-414. doi: 10.4269/ajtmh.2000.62.413.
16. Zar JH. Biostatistical Analysis. 4^th^ ed. Hoboken: Prentice Hall. 1999.
17. Tiawsirisup S, Platt KB, Evans RB, Rowley WA. Susceptibility of *Ochlerotatus trivittatus* (Coq.), *Aedes albopictus* (Skuse), and *Culex pipiens* (L.) to West Nile virus infection. Vector Borne Zoonotic Dis. 2004;4: 190-197. doi: 10.1089/vbz.2004.4.190.
18. Tiawsirisup S, Platt KB, Evans RB, Rowley WA. A comparison of West Nile Virus transmission by *Ochlerotatus trivittatus* (COQ.), *Culex pipie*ns (L.), and *Aedes albopictus* (Skuse). Vector Borne Zoonotic Dis. 2005;5: 40-47. doi: 10.1089/vbz.2005.5.40.
19. Komar N, Dohm DJ, Turell MJ, Spielman A. Eastern equine encephalitis virus in birds: relative competence of European starlings (*Sturnus vulgaris*). Am J Trop Med Hyg. 1999;60: 387-391. Doi: 10.4269/ajtmh.1999.60.387
20. Marchi R, Nagaswami C, Weisel JW. Fibrin formation and lysis studies in dengue virus infection. Blood Coagul Fibrinolysis. 2009;20(7): 575-582. doi: 10.1097/MBC.0b013e32832fb1cf.
21. Kent R, Juliusson L, Weissmann M, Evans S, Komar N. Seasonal blood-feeding behavior of *Culex tarsalis* (Diptera: Culicidae) in Weld County, Colorado, 2007. J Med Entomol. 2009;46: 380-390. doi: 10.1603/033.046.0226.
22. Golnar AJ, Turell MJ, LaBeaud AD, Kading RC, Hamer GL. Predicting the mosquito species and vertebrate species involved in the theoretical transmission of Rift Valley fever virus in the United States. PLoS Negl Trop Dis. 2014;8(9): e3163. doi: 10.1371
23. Kilpatrick AM, LaDeau SL, Marra PP. Ecology of West Nile virus transmission and its impact on birds in the western hemisphere. The Auk. 2007;124: 1121-1136.
24. Dohm DJ, O’Guinn ML, Turell MJ. Effect of environmental temperature on the ability of *Culex pipiens* (Diptera: Culicidae) to transmit West Nile virus. J Med Entomol. 2002;39: 221-225. doi: 10.1603/0022-2585-39.1.221.
25. Apperson CS, Hassan HK, Harrison BA, Savage HM, Aspen SE, Farajollahi A, *et al*. Host feeding patterns of established and potential mosquito vectors of West Nile virus in the eastern United States. Vector Borne Zoonotic Dis. 2004;4: 71-82. doi: 10.1089/153036604773083013.
26. Kilpatrick AM, Kramer LD, Jones MJ, Marra PP, Daszak P. West Nile virus epidemics in North America are driven by shifts in mosquito feeding behavior. PLoS Biol. 2006;4: e82. doi: 10.1371/journal.pbio.0040082.
27. Molaei G, Andreadis TG, Armstrong PM, Anderson JF, Vossbrinck CR. Host feeding patterns of *Culex* mosquitoes and West Nile virus transmission, northeastern United States. Emerg Infect Dis. 2006;12: 468-474. doi: 10.3201/eid1203.051004.
28. Levine RS, Mead DG, Hamer GL, Brosi BJ, Hedeen DL, Hedeen MW, *et al*.. Supersuppression: Reservoir competency and timing of mosquito host shifts combine to reduce spillover of West Nile virus. Am J Trop Med Hyg. 2016;95: 1174-1184. doi: 10.4269/ajtmh.15-0809.

**Supporting information**

**S1 Table 1.**
