# Supplementary material for: Bird species define the relationship between West Nile viremia and infectiousness to Culex pipiens mosquitoes
Source: PLoS Negl Trop Dis. 2022 Oct 6;16(10):e0010835. doi: 10.1371/journal.pntd.0010835 (PMC9578590; doi:10.1371/journal.pntd.0010835)
Supplement: Attachment — Submitted filename: PLoS_2022-Response Letter-2.docx [file pntd.0010835.s004.docx]

RESPONSE TO REVIEWER COMMENTS / SUGGESTIONS

GENERAL COMMENTS

The comments and suggestions offered by the reviewers are greatly appreciated. In response, the manuscript has undergone extensive revision. The most important and expansive revisions include:

- Additional data analyses have been performed to include logistic regression modelling with a random effect variable, as recommended by Reviewer 3. To do this, we enlisted a colleague in the UND Biology Dept., Professor Robert A. Newman, who teaches undergraduate and graduate level classes in population modeling and uses modeling in his own research. Dr. Newman is now included as an author. A new figure and table showing the results of this analysis are included in the revised manuscript (Figure 1). We have also recalculated the host competence index values.
- The Introduction section is completely rewritten and condensed as suggested by the reviewers. Text describing examples of viremia-mosquito infectiousness by other scientists has been moved to the Discussion section.
- The Supporting Information section is greatly expanded to address many of the reviewers’ concerns. This section now presents details on
  - weight/sex of birds,
  - how host viremias were determined,
  - the raw data, noting which infectious feeds were excluded from final analysis and why,
  - the method used to calculate reservoir competence index values.

_________________________________________________________________

RESPONSE (indicated by blue font) TO COMMENTS – **REVIEWER 1**

1. Were the birds tested daily for viremia? How was the duration of infectiousness determined.? With HOSP hosts, mosquitoes can be infected for up to 2 wks post infection despite the waning viremia.

Birds were tested for viremia for three consecutive days after being inoculated with virus. The duration of the birds’ infectiousness was determined by feeding fresh batches of mosquitoes on the birds on each consecutive day.

2. What was the sex and age of the host birds?

This information is presented in the expanded Supporting Information, Table S-1.

3. It was unclear why some birds with a microfilaremia affected mosquito infection while others did not.

Only one microfilaremic bird (Grackle #42) affected WNV infection in the mosquitoes. This bird exhibited a very high viremia. Experiments described in our earlier paper [21] indicate that WNV does not adhere to the surface of grackle microfilariae (*Chandlerella quiscali*). As a result, grackle microfilariae do not transport the virus on their surface as they penetrate the mosquito midgut, as is the case with *Brugia* microfilariae and alphaviruses. Thus, an extremely high viremia is required in order for microfilarial enhancement to occur in the grackle-WNV-*Culex pipiens* transmission system.

4. How were the birds anesthetized? What was used?

This information has been added to the Materials and Methods section, lines 71 – 75.

5. Unclear why the now essentially extinct NY99 strain was used in these experiments.

The original hypothesis of the investigation was that pre-existing microfilaremia in passerine birds may have promoted the rapid spread of WNV from New York across the Midwest after its introduction into North America. Hence the choice of the original WNV strain.

Results -This section was somewhat tedious, but generally well done.

The Results section has been extensively re-written with the goal of being less tedious to read.

Conclusions – In general the data were clearly presented, appropriately analyzed and support the conclusions.

Thank you.

Minor comments have been inserted on the attached manuscript using tracked changes.

This have been incorporated into the revision.

1. Literature review. The authors have missed quite a few papers where mosquito infection was used to compare avian host competence, making their current study less unique than described in the Introduction.

Additional references have been cited. The number of references has increased from 28 to 46.

2. Introduction. I think too much space is given to a review of papers showing methods of mosquito in vitro infection can alter vector competence results. The focus should be on in vivo information as this pertains to the experiments at hand.

The review of papers showing methods of mosquito *in vitro* infection have been moved to the Discussion. Inclusion of these works is germane and supports the findings of this report because those *in vitro* experiments show unequivocally that by keeping virus concentration constant but changing the milieu in which the virus is suspended can significantly CHANGE the mosquito infection outcome. This is what we have observed using live viremic hosts.

2. Conclusions. The dynamics of WNV transmission is complex, as shown in the current paper where there were differences seen between pipiens colonies and host bird species. Therefore, I think that generalizations used for gross comparisons still are useful and I wouldn't throw out the Komar host competence index just yet. As used by Kilpatrick and Wheeler et al. it still provides a useful way of comparing avian species.

We agree, the Komar host competence index will be useful for many years to come. That is not in dispute. This report only suggests that the index can and should be made more precise for high-priority host species by conducting actual infectious feeds with those high-priority host species rather than relying on infection data from a surrogate model system that may or may not reflect reality.

____________________________________________________________________

RESPONSE (indicated by blue font) TO COMMENTS – **REVIEWER 2**

1. Overall, the paper is very well written.

Thank you.

2. Several different types of units are used for describing virus concentration. TCID50, IC50, LC50, pfu, etc. These need to be reviewed and clarified. At a minimum, spell out abbreviations when first used.

Unfortunately, different measures of virus concentration were used in different studies. However, as suggested, we have written out the each of these the first time they were used.

3. The capitalized name of a bird species refers to the species as a whole, not individual animals. Therefore, these should not be pluralized. There is only one American Robin, but there can be multiple American robins. Also be consistent with capitalization of bird names. In the Intro, you do not capitalize house finch.

We have corrected the capitalization of the bird species throughout, as suggested.

4. In the second paragraph of the introduction, treatment of vertebrate reservoir competence is confusing because you are actually referring to reservoir capacity rather than competence. Competence describes the potential to infect mosquitoes, whereas capacity measures the relative importance of different species as amplifiers. The background on transmission dynamics can probably be streamlined into a single paragraph. If necessary, further description of the processes involved could be added to the discussion.

The terminology regarding competence vs. capacity has been cleaned up throughout the revised manuscript. As noted above, the Introduction section has been completely rewritten and description of host competence index values has been moved to the Discussion section (paragraphs 4 – 6, lines 323-355).

5. The Intro is too heavy on background information and too light on presenting the specific scientific problem to be addressed and a hypothesis statement. Please add a hypothesis to the final paragraph in the Introduction.

Agree. This has been done and the goal of the study is stated (lines 62-64).

6. Why were American Robin and Common Grackle chosen for this study? Was there prior evidence that the blood of these two species differed with respect to WNV infectiousness?

The original impetus of the investigation was to understand if pre-existing microfilaremia in passerine birds enhanced transmission of WNV. The American Robin is one of the most important amplifying hosts of WNV. The Common Grackle is also susceptible to WNV and there is more known about avian filariasis in the Common Grackle than any other passerine species. Both bird species are common, synanthropic “backyard” species within the upper Midwest, a region in which WNV has among the highest human incidence in the nation.

7. When presenting average viremia values, do these represent the log of the mean, or the mean of the logs? In other words, do you log transform for presentation purposes before or after calculating the mean value? It makes a difference. This should be explained in the methods.

We converted the titers to log titers and then averaged them. Note, to determine viremias, titers were only averaged for individual birds for individual feedings. They were not averaged over time or for multiple birds. A detailed explanation of how host viremias were determined is provided in the beginning narrative and Table S-1 in the Supporting Information.

8. Why are you including the data for the Rutgers mosquito strain?? Do these data add to the quality of the experiment?

We believe that inclusion of a second strain of mosquitoes supports the applicability of our findings to wild populations of this mosquito species. Because the same trend was observed in both mosquito strains, the differences in host infectivity between robins and grackles is therefore more likely to be a real phenomenon rather than a laboratory artifact.

9. Supplemental data file is provided, but I did not notice a citation or reference to these data in the main text.

A greatly expanded Supporting Information file has been included and is referenced in the text where appropriate (e.g., see lines 106, 136, 151, etc.).

***Specific comments***:

Line 138 quiscula is misspelled.

Thank you for pointing this out. It has been corrected.

Line 167-169. In order to make your study repeatable by a reader, please explain how you converted three engorged mosquitoes into a viremia measure. Did you measure the volume of blood in each mosquito? Were the mosquitoes each tested in order to produce a mean viremia determination?

Yes, each engorged mosquito was tested individually and the titers obtained averaged. A detailed explanation of how host viremias were determined is provided in the beginning narrative and Table S-1 in the Supporting Information.

Line 169-170. Why were mosquitoes removed for testing on four different days post-feeding? Was there a difference for mosquitoes that incubated for 3 days versus 7 days?

Again, the original impetus for this study was microfilarial enhancement, a process that accelerates virus dissemination and shortens the extrinsic incubation period. To observe this, we tested nearly all mosquitoes after a very short extrinsic incubation period – much sooner than we would normally would. We did not observe a noticeable difference in infection rates due to sampling day.

Line 260 Use “index values” rather than “indices”. You are discussing multiple values within a single index. Also there are probably some fancy statistics to generate confidence intervals around the index values, and a p-value for the comparison. I also question the precision of these values. Is reporting out to three significant figures appropriate for any of the measures in this Table?

Agree, the term “index values” has been substituted for the term “indices”. We have presented confidence intervals (details on their computation area described in Supporting Information). The index values are now presented out to 2 decimal places.

Line 286 Fig 1 can be gray-scale. Color not required.

Agreed. Figure 1 now is Figure 2. It has been re-drawn and is now wholly black-and-white.

Line 302 dog/horse versus cattle/sheep sera.

This has been fixed (see line 318).

Line 363. And vector species, for that matter.

Yes! The importance of the vector species has always, since the days of Walter Reed, been recognized as integral to the relationship between host viremia and mosquito infection. The importance of vector species is stated up front in the Introduction of the revised manuscript (lines 58-61).

____________________________________________________________________

RESPONSE (indicated by blue font) TO COMMENTS – **REVIEWER 3**

The following **major issues** were identified in a bulleted list on the first 1 ½ pages of the critique:

This report only discusses infection, not dissemination or transmission.

Yes, the reviewer is correct that infection “is the least epidemiologically important metric of vector competence”. But it is also biologically true that disseminated infection and oral transmission will not occur without infection. So in a sense, this report represents the beginning, not the conclusion, of a new line of inquiry. As indicated above, the original impetus for this study was microfilarial enhancement, a process that accelerates virus dissemination and shortens the extrinsic incubation period. To observe this, we tested nearly all mosquitoes after a very short extrinsic incubation period – much sooner than we would normally would. Because mosquitoes were sampled so early, data were not suitable for analyzing normal viral dissemination in these mosquitoes.

If grackles are an anomaly, then these results are of minor importance.

Perhaps. But at this point, no one knows. And we will never know until more bird species are tested, using direct mosquito feedings on live, viremic birds. That very plea is made in the Abstract, the Author Summary, and the next-to-last paragraph of the Discussion.

The use of highly inbred mosquitoes may lead to spurious conclusions.

Yes, but there were two separate lines of highly inbred mosquitoes that showed essentially the same trend – robins with lower viremia were more infectious than grackles with higher viremia. This provides a measure of confidence that the trend may occur also with wild mosquitoes.

The viremia data aren’t shown.

The Supporting Information section has been expanded to include details on the viremias and how they were determined.

The link to published literature is very poor.

The number of references has increased from 28 to 46.

Statistical analysis does not account for repeated feedings on the same bird.

To address this deficiency, we enlisted a colleague with expertise in statistics and modelling to perform the requested analyses (*i.e*., logistic regression with random effect variable [birdID] included). He has been added as a co-author to the revised manuscript.

RESPONSE TO COMMENTS – **REVIEWER 3** – SPECIFIC COMMENTS

Twenty-seven specific comments were listed on page 2 to 5 of the critique

L36 Why “peak viremia”? Do you mean “viremia”? In the abstract you don’t give any reason for the infectiousness to be different for different birds. Is this possibly related to filarial worms or other things?

This sentence has been changed (lines 29-30). No, only in one bird (Grackle #42) were microfilariae involved in altering host infectiousness to mosquitoes. Data from that individual bird have been excluded from the analysis for this report.

L46-63 There isn’t a single reference in the entire first paragraph. It’s also a very basic first paragraph that belongs in an introductory textbook, not a primarily literature paper. I’d strongly suggest deleting almost the entire paragraph and starting the paper off with a statement of the research gap that the paper is filling by introducing the reader to the vast vast literature on this topic. For every relevant sentence in this paragraph there are literally dozens of citations that would be useful to give the reader to introduce them to the previous science on this topic.

The Introduction has been shortened considerably, as suggested by other reviewers.

L56-7 This text makes it seem like vectors either transmit or don’t when, in fact, it is a continuous trait with most (but not all) species being able to transmit a little with high enough viremia and the right temperature and time. These species are very weakly competent vectors. Others are moderately competent and some are, relatively speaking, highly competent. But I’ve never seen any data showing any species that is 100% competent in that it always transmits after feeding on low viremias regardless of the temperature post feeding or the time post feeding. You are doing the reader a great disservice explaining this basic concept this way. See comment above – I’d delete almost this entire paragraph anyway.

Done.

L64 The claim of “most important” isn’t supported by data or citations here. One could easily refute this argument by showing that transmission following infection is zero at low temperatures. Remove this unsupportable claim and add several references to give the reader a way to see previous work on this topic.

Done.

L68-70 Like the description above of “vectors” this categorization of hosts and “amplifying” or “dead- end” is again an oversimplification of a continuous trait into two categories that badly misleads the reader. Are European starlings “amplifying hosts”? Komar et al 2003 EID showed that they have viremias that are barely infectious and thus would rarely lead to mosquitoes that would transmit, but a very tiny fraction of mosquitoes would, if they fed on them on their day of peak viremia and were held at the right temperature for the right amount of time. Similarly, Rock doves were assigned a host competence value of 0 by Komar et al 2003 EID suggesting they were dead end hosts. Later work showed that with a larger sample of birds that were sampled in the field, some were weakly competent. If you are going to explain this basic concept, do so correctly and indicate that host competence is a continuous trait with different fraction of mosquitoes becoming infected and going on to transmit and that this depends not just on host viremia but also temperature, the day they are tested, whether they were food stressed as larvae, etc.

The Introduction has been shortened considerably (as suggested by other reviewers) and this line of text has been removed.

L68-70 There is huge variation among birds in viremias, so a single bird species doesn’t have a single competence value. Some individual birds within a species are highly infectious whereas others are quite poor.

This line of text has been removed.

L75 This is the first citation in the paper. By this time you should have cited 10-20 other papers.

The Introduction has been shortened considerably.

L121 The year for Ref 10 is incorrect. It is a 2005 paper, not 2004.

Thank you for catching this error. It has been corrected.

L122-127 I went back and read Ref 10 to examine this case and you’ve neglected to mention that the populations of mosquitoes feeding on the finch and the chicken are from different places in Kern county so it’s not surprising at all that the fraction infected differed. Work by both Laura Kramer’s lab (doi:10.4269/ajtmh.2010.10-0005) and Bill Reisen’s group (<https://doi.org/10.1093/jmedent/45.6.1126>) showed that vector competence for a single species of mosquito can vary substantially over time and over short distances. In fact, none of the feedings of both chicks and finches in ref 10 used mosquitoes from the same population of mosquitoes so this study cannot be used to make claims about differences among species in infectivity to mosquitoes with the same viremia.

True, those mosquitoes were not collected at the same site, but they did come from the same county, Kern Co. CA. And the *Culex tarsalis* populations in Kern Co. and surrounding areas have been shown in two separate studies to be panmictic. So, there was probably a reasonable amount of genetic similarity in the two populations of *Cx. tarsalis* used in the Reisen et al. 2005 study. Plus, the difference in infection rate between the mosquitoes fed on viremic chicken (13%) versus viremic finch (94%) was so great that it seems improbable that there was not some sort of “host species effect” at play. To make this more precise, we have added the word “panmictic” in the sentence (line 301).

L144-145 24 hr is an extremely short acclimatization period. Most studies with wild birds use 2 weeks!

The dates of capture and the dates of mosquito feeds for each bird is provided in the Supporting Information, Table S-1. This information indicates that birds were acclimatized to captivity for a period of 2 ½ weeks (robins) up to 2 ½ years (Grackle #102).

L156-8 These are highly inbred lines of mosquitoes. It’s not clear to me that the results from these mosquitoes tell us much about how wild mosquitoes would perform in the same experiments.

We believe that inclusion of a second strain of mosquitoes supports the applicability of our findings to wild populations of this mosquito species. Because the same trend was observed in both mosquito strains, the differences in host infectivity between robins and grackles is therefore more likely to be a real phenomenon rather than a laboratory artifact

L173 Why did you only test for infection? This is the least relevant of measures of vector competence. Salivary secretions would be most relevant but disseminated infections would at least demonstrate escape from the midgut. Analysis of the disseminated infection data in the supplemental material produce very different results (see below for analysis).

True, we tested for dissemination (i.e., legs) in mosquitoes whose bodies tested positive. But the original object of the sampling was to look for *EARLY* dissemination due to microfilarial enhancement. Therefore, nearly all mosquitoes were tested after a very short extrinsic incubation period – much sooner than we would normally would. Because mosquitoes were sampled so early, these data are NOT suitable for analyzing normal viral dissemination.

L165-171 What temperature were mosquitoes held at?

26^o^ C. This has been added to the Materials & Methods, subheading “Mosquito Infections”.

L179 Why not simply fit all the data to a GLM with a binomial distribution with species and viremia and species*viremia as predictors? This would answer the question in a much simpler way. In addition, since there are repeated feedings on the same birds the analysis must account for this and is easiest done with a mixed effects model (e.g. glmer). See below for this analysis.

Agree. The statistics have been completely re-done.

L187 It’s not clear how the statistics were done here, but a simple comparison suggests something is badly wrong. If the two slopes are 0.25 and 0.58 and the pooled SE of these values is ~0.09, then the t-value should be (0.58-0.25)/0.09 = 3.66. Given the very large difference in slopes relative to the SEs of the slopes it is definitely NOT valid to combine data from the two strains of mosquitoes. What strains of mosquitoes were fed on the robins?

Agree. The slopes are compared by comparing respective 95% confidence intervals. Both strains of *Cx. pipiens* were fed on both robins and grackles (see Table S-2 in Supporting Information).

L197-8 Which viremia are you using? An average? If the relationship isn’t linear, then an average isn’t appropriate. Further, what do you consider an infectious viremia? One that results in 1% of mosquitoes becoming infected? With the sample sizes you’ve used, you can’t exclude low fractions of mosquitoes being infected.

For analysis, we used the viremia for a given bird at a given feeding. These were not averaged across time or between birds. Determination of viremias is described in detail in the Supporting Information, Table S-1.

L202 What is the IACUC protocol number covering this research?

The UND IACUC protocol number has been added (line 143). As in all previous publications, USAMRIID does not allow publication of individual IACUC protocol numbers.

L211 You absolutely must show the viremias in this paper since it the whole focus of the study.

Individual viremias for all infectious feeds are provided in the Supporting Information, Table S-1.

L222 This isn’t justified. See comments above.

Sorry, but we do not understand this comment. We wanted to know if there was a difference in viral susceptibility between two strains of *Cx. pipiens*. Therefore, if the two strains fed on the same bird at the same time, we compared them. Likewise, if the two strains fed on two different birds of the same species, but with identical viremias, we compared them. Why is this not appropriate?

L225-234 The analysis of this data is very messy and problematic. Instead, as suggested above, the authors should do a single GLM with the whole dataset with a binomial distribution and predictors for bird species, mosquito strain, viremia and interactions which will avoid arbitrary post-hoc combining of data. See below for this analysis that also includes random effects to account for repeatedly feeding on the same bird.

Data analysis has been re-done as suggested.

L260 Why are the “days of infectiousness” so low? The data in Komar et al 2003 EID suggest that both robins and grackles are partly infectious for at least 4 days.

This is true, the competence index values for grackles cannot be directly compared between this study and that of Kilpatrick *et al*. [42] because the Kilpatrick *et al*. index value was based on viremia parameters established for grackles by Komar *et al*. 2003. In that study, grackles (n=6) displayed a much higher, longer-lasting viremia. A possible explanation is that in the Komar *et al*. study, grackles were infected by multiple (up to 15) infectious mosquito bites whereas in this study, grackles were infected by needle inoculation. Previous studies (*e.g*., Styer et al. 2001, 2006; Moser et al. 2015) have demonstrated that WNV infections induced by infectious mosquito bites can produce higher viremia than infections induced by needle inoculation.

L267 Where are these 95% CIs? Need to refer to Table 4 here.

The Table 4 in previous version has been removed. The 95% CL are presented in Figure 2 of the revised manuscript.

L279 What is the time point for all these analyses? How many days after feeding on the bird?

Mosquitoes were tested 4, 5, or 7 days after feeding on a viremic bird.

L286 This way of presenting the data is very non-intuitive for the reader. Why not show the fraction of mosquitoes infected? The data are 0/1 and thus a logistic regression is more appropriate theoretically. See below for plots of the data and fitted models that are much easier to understand.

Data analyses were re-done as suggested.

L336-7 This comparison is completely misdirected. The key difference between the competence estimates in the current study and that of ref 23 is not the assumption that a given viremia produces the same infectiousness. The key difference is that the viremias used in ref 23 come from Komar *et al* 2003 EID (which should be cited), in which viremias for grackles were 3-5 log10 higher than for robins, including a very high viremia on day 4 post-infection. The data in Fig 1 (and ref 14) suggests that in this study viremias were similar or slightly lower for grackles. So, the main difference in competence values is only partly due to differences in the viremia-infectiousness relationship, and moreso due to differences in viremias observed in the experimental infections.

Agree. The former comparison has been discarded. In its place, we have computed host competence index values only for our infections. The comparison is no longer between our computations and those of Kilpatrick *et al*., rather the comparison is made between computations done with actual robin/grackle infections versus calculations done with the surrogate chicken infection model. Details on how we did this are in Supporting Information.

L351-3 This sentence is very strange in ignoring the last decade of work on West Nile virus. A half dozen studies have now shown that neither highly viremic hosts (crows, jays), nor common birds (house sparrows) play an important role in feeding mosquitoes. Instead, hosts that are highly preferred by feeding mosquitoes (primarily robins) are more important than all these species (often combined) and that these species are often uncommon (making up <10% of the birds at a site). All these papers should be cited. However, more broadly, in order to determine which hosts are important in infecting mosquitoes, it’s critical to be able to quantify how infectious each species is. The work here suggests that more work needs to be done to determine if there is substantial differences among species in their infectiousness given the same viremia or if grackles are simply anomalous.

This section has been modified appropriately.

L355 This is incorrect. Robins are not particularly abundant. See comment and papers above. They are often relatively uncommon.

Come to eastern North Dakota during the summer and you may change your opinion about robin population abundance. So many robins, so much WNV in these parts.

**A note on Revised Statistical analyses**

On pages 6 – 11, the Critique from Reviewer displays R model statements and statistical results (*i.e*., 6 tables and 2 graphs) from analyses of the data provided in Supporting Information of the original manuscript.

This was a problem that is totally our fault.

There were two data sources that SHOULD HAVE BEEN EXCLUDED from that analysis. Grackle #42 (all data) and Robin #108 (day 2 viremia, Rutgers strain):

1. Grackle #42 displayed microfilarial enhancement and therefore, NONE of the infection data from this bird should have been included in the data analysis.
2. Also, there was one infection identified as a statistical outlier – Robin #108, Day 2 w/ the Rutgers strain of mosquito. The following table shows the infection history of that bird.

|  |  | Day 1 | Day 2 | Day 3 |
| --- | --- | --- | --- | --- |
| Mosquito Infection Rate (n) |  | log_10_ 7.0 | log_10_ 7.3 | log_10_ 4.3 |
|  | Rutgers | 100% (23) | 71% (21) | Not tested |
|  | Area B | 100% (11) | 91% (11) | Not tested |

*Test for Outlier*. Three other robins had viremias between 7.0 and 7.4 (as did Robin 108 on Day 1). All these infected >90% of the Rutgers strain of mosquitoes that fed on them, suggesting that Robin 108, day 2, Rutgers could be an outlier. Using excel software, we computed the interquartile range for this set of five infection rates, multiplied by 1.5, and subtracted that value from the first quartile to determine the minimal cut-off – i.e., 78.6%, confirming that the data point in question (71%, see shaded area in table above) was a statistical outlier. Therefore, those data (*i.e*., the 21 Rutgers strain mosquitoes fed on Robin 108, day 2) should NOT have been included in the data analysis.

With the data from those two infections excluded (*i.e*., a total of 120 mosquitoes), the logistic regression analyses were repeated using model statements almost exactly as recommended by Reviewer 3. The results of those analyses are presented in Figure 1 of the revised manuscript.
